# Hub Genes Identification, Small Molecule Compounds Prediction for Atrial Fibrillation and Diagnostic Model Construction Based on XGBoost Algorithm

**DOI:** 10.3389/fcvm.2022.920399

**Published:** 2022-07-14

**Authors:** Lingzhi Yang, Yunwei Chen, Wei Huang

**Affiliations:** Department of Cardiology, The First Affiliated Hospital of Chongqing Medical University, Chongqing, China

**Keywords:** atrial fibrillation, Connectivity map, the eXtreme Gradient Boosting algorithm, the Sharpley Additive exPlanations, rank robust aggregation, weighted gene coexpression network analysis

## Abstract

**Background:**

Atrial fibrillation (AF) is the most common sustained cardiac arrhythmia and engenders significant global health care burden. The underlying mechanisms of AF is remained to be revealed and current treatment options for AF have limitations. Besides, a detection system can help identify those at risk of developing AF and will enable personalized management.

**Materials and Methods:**

In this study, we utilized the robust rank aggregation method to integrate six AF microarray datasets from the Gene Expression Omnibus database, and identified a set of differentially expressed genes between patients with AF and controls. Potential compounds were identified by mining the Connectivity Map database. Functional modules and closely-interacted clusters were identified using weighted gene co-expression network analysis and protein–protein interaction network, respectively. The overlapped hub genes were further filtered. Subsequent analyses were performed to analyze the function, biological features, and regulatory networks. Moreover, a reliable Machine Learning-based diagnostic model was constructed and visualized to clarify the diagnostic features of these genes.

**Results:**

A total of 156 upregulated and 34 downregulated genes were identified, some of which had not been previously investigated. We showed that mitogen-activated protein kinase and epidermal growth factor receptor inhibitors were likely to mitigate AF based on Connectivity Map analysis. Four genes, including *CXCL12, LTBP1, LOXL1*, and *IGFBP3*, were identified as hub genes. *CXCL12* was shown to play an important role in regulation of local inflammatory response and immune cell infiltration. Regulation of *CXCL12* expression in AF was analyzed by constructing a transcription factor-miRNA-mRNA network. The Machine Learning-based diagnostic model generated in this study showed good efficacy and reliability.

**Conclusion:**

Key genes involving in the pathogenesis of AF and potential therapeutic compounds for AF were identified. The biological features of *CXCL12* in AF were investigated using integrative bioinformatics tools. The results suggested that *CXCL12* might be a biomarker that could be used for distinguishing subsets of AF, and indicated that *CXCL12* might be an important intermediate in the development of AF. A reliable Machine Learning-based diagnostic model was constructed. Our work improved understanding of the mechanisms of AF predisposition and progression, and identified potential therapeutic avenues for treatment of AF.

## Introduction

Atrial fibrillation (AF) is the most common type of arrhythmia, with major public health implications and increasing prevalence (1). Currently, the treatments for AF mainly includes rhythm control, rate control, and antithrombosis (2). Although progress has been made in treatment of AF, current therapy strategies have important limitations (3), including adverse effects risk, incomplete efficacy, and a significant long-term recurrence rate (4). Therefore, an improved understanding of the pathogenesis of AF and atrial substrate remodeling is necessary for development of novel therapeutic approaches and new management strategies.

Screening and detection of AF are complex due to its latent and asymptomatic properties. A clinical decision support system for diagnosis and prediction of prognosis is needed. Machine learning has been widely used to assist decision making and model construction. The eXtreme Gradient Boosting (XGBoost) (5) strategy is a popular and effective approach for classification, and its efficacy has been widely validated in lots of diseases. For example, Ogunleye et al. designed an accurate diagnostic model for chronic kidney disease (CKD) using the XGBoost method (6), which showed satisfactory performance.

The purpose of this study was to identify key genes, pathways, potential therapeutic drugs, and underlying regulatory networks of hub genes related to AF. The hub genes would then be used to construct a diagnostic model to provide tools for clinical practice. Transcriptomic microarray datasets of AF patients were extracted and bioinformatic methods were used to screen for robust candidate genes. Potential therapeutic targets and small molecule compounds were predicted. Using these approaches, we developed a comprehensive understanding of the role of microenvironmental immune regulation of CXCL12 in AF, and suggested that CXCL12 might be a marker for distinguishing AF subsets. This provided new insights into the mechanisms of AF and identified potential therapeutic agents for management of AF. Furthermore, a reliable diagnostic model was constructed for AF using the XGBoost algorithm.

## Materials and Methods

### Microarray Datasets

Gene Expression Omnibus (GEO)^1^ was used to search datasets of patients with AF. To identify relevant GEO datasets relevant to differences between patients with AF and sinus rhythm (SR), we used the following keywords: atrial fibrillation OR atrial flutter. In addition, the reference lists of relevant articles and reviews were manually searched to ensure the completeness of the literature search.

The inclusion criteria were as follows: (1) Gene expression data from the atrium, the atrial appendage, or the sleeve of the pulmonary vein tissue from individuals with AF and individuals with SR; (2) Data that could be reanalyzed.

**FIGURE 1:**
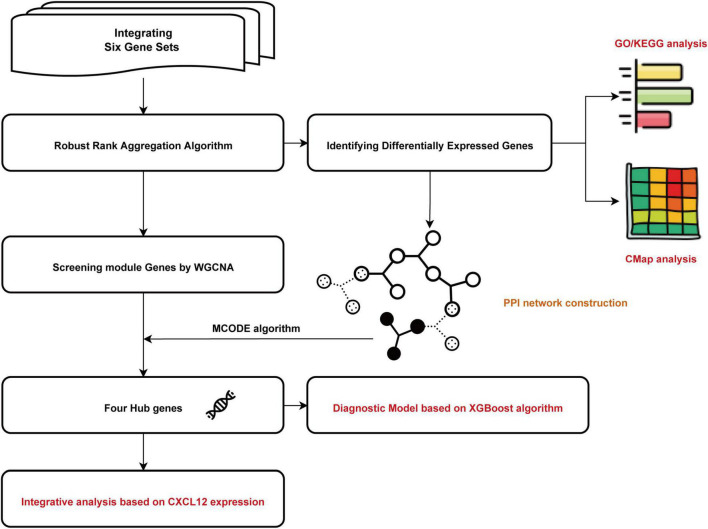
Flowchart of the bioinformatics analysis. GO, Gene Ontology; KEGG, Kyoto Encyclopedia of Genes and Genomes; WGCNA, the weighted gene coexpression network analysis; MCODE, Molecular Complex Detection; CMap, Connectivity Map. XGBoost, the eXtreme Gradient Boosting.

All relevant manuscripts were independently reviewed by two investigators (LY and YC) to identify whether the studies met the inclusion criteria. The workflow for bioinformatic analyses is shown in Figure 1.

### Robust Rank Aggregation Analysis and Integration of Datasets

Gene expression profiling was annotated using the corresponding annotation packages and R software. All Affymetrix data were normalized using the justRMA function (7). We performed RRA analysis to identify robust differentially expressed genes (DEGs) using the R package “Robust Rank Aggregation” (8). In the final list, genes with RRA scores less than 0.05 were selected as DEGs.

### Functional Enrichment Analysis and Protein–Protein Interaction Analysis

We performed GO^2^ and KEGG enrichment analyses^3^ using the “clusterProfiler” package (9) in R software to match the biological themes of the gene clusters (threshold of adjusted *p* < 0.05). The STRING database^4^ was used to establish a PPI network. To identify hub genes, the Molecular Complex Detection (MCODE) plugin was used in Cytoscape software (version 3.9.0).

### Weighted Gene Co-expression Network Analysis

Genes with *p* values < 0.05 and logarithmic fold changes (logFCs) > 0.25 were selected from RRA results to perform WGCNA. To increase the number of samples and improve the reliability of the results, we integrated and normalized the six datasets by batch normalization using “sva” (10) and “limma” package (11) in R software. Key modules were identified by setting the soft-thresholding power to 8 (scale-free R2 = 0.83), cut height to 0.25, and minimal module size to 30.

### Connectivity Map Analysis

Connectivity map (CMap)^5^ analysis (12) was used to identify potential compounds that perturbated AF expression signature. Mechanisms of action (MoA) analysis of the top 50 compounds was performed to identify the shared mechanisms of action of these compounds.

### Integrative Analyses of the Key Gene *CXCL12*

We performed receiver operating characteristic curve (ROC) analysis to investigate the classification capacity of identified genes. We chose *CXCL12* for further analysis because its area under curve (AUC) was the highest among the hub genes (Supplementary Figure 1). The expression of *CXCL12* was determined according to the quantile value for the AF patient cohort. The “limma” package was used to obtain DEGs between the high *CXCL12* and low *CXCL12* AF subgroups (threshold of adjusted *p* < 0.05 and logFCs > 2).

### Gene Set Enrichment Analysis and Single-Sample Gene Set Enrichment Analysis

Gene set enrichment analysis (GSEA) is a widely used computational method to determine whether an *a priori* defined set of genes is significantly differentially expressed between two biological states. We performed GSEA on gene sets with high and low *CXCL12* expression to explore the biological function of *CXCL12* in AF.

The single-sample GSEA (ssGSEA) method is an extension of the GSEA method used to analyse a single sample. We used ssGSEA to estimate the infiltration levels of 28 immune cell types in the high *CXCL12* and low *CXCL12* groups.

### Immune Cell Infiltration Analysis

A deconvolution algorithm developed by Newman et al. (13) called “CIBERSORT” was used to estimate of the abundances of different cell types in a mixed cell population. We scored 22 immune cell types based on their relative abundances in AF samples. Differences in infiltration of immunocytes between the high *CXCL12* and low *CXCL12* groups were analyzed using Spearman correlation and the Wilcoxon rank-sum test.

### Construction of a TF-miRNA-mRNA Network

We downloaded the microRNA expression dataset GSE28954 (14) and identified differentially expressed miRNAs (DE-miRNAs) between individuals with AF and individuals with SR using the “limma” package in R software with adjusted *p* < 0.05 as the threshold. Then, miRNet 2.0^6^ (15) was used for construction of the possible transcription factor (TF)-miRNA-mRNA network.

### Physicochemical Properties Analysis

Human Protein Atlas^7^ was used to explore the cellular location of *CXCL12*, followed by ProtParam, ProtScale^8^, and TMHMM 2.0^9^ to analyze the physicochemical properties of CXCL12 and to predict transmembrane helices in CXCL12, respectively.

### Diagnostic Model Construction Using a Machine Learning Algorithm

This study used the XGBoost algorithm to develop diagnostic model. XGBoost is an integrated learning algorithm based on boosting algorithms. Integrated learning uses a selected method to learn multiple weak classifiers with differences, followed by combination of these classifiers. ROC analysis of the diagnostic model was performed using the pROC package (16). The model was subjected to internal validation using the bootstrap method (17) with 1,000 iterations. The Brier score for the diagnostic model was calculated.

### Sharpley Additive exPlanations Interpretation Method

To interpret and understand the features of genes from the diagnostic model, we used the Sharpley Additive exPlanations (SHAP) interpretation method to explain the XGBoost classification result, which allowed for analysis of each feature. Jupyter notebook was used to visualize these results.

### Statistical Analysis

Statistical analyses and data visualization were performed using R software and Jupyter notebook. Spearman’s correlation analysis was performed to estimate the correlation between different immune cells and Wilcoxon rank-sum test was used to estimate the differences between two groups. Associations were considered as statistically significant at two-sided *p*-values < 0.05.

## Results

### Atrial Fibrillation Microarray Datasets

After filtering the GEO database, six AF microarray datasets were selected. The basic information associated with these GEO datasets is listed in Table 1. The number of patients with AF in each study ranged from 4 to 32, and the number of controls ranged from 2 to 31. A total of 93 patients with AF and 76 controls were included.

**TABLE 1:** Summary of the six expression datasets involved in this study.

GSE accession	Platform	Total number (SR:AF)	Tissues
GSE2240 (74)	GPL570	30 (20:10)	Atrium
GSE14975 (75)	GPL570	10 (5:5)	Atrium
GSE41177 (76)	GPL570	38 (6:32)	Atrial appendage or sleeve of pulmonary vein tissue
GSE79768 (77)	GPL570	26 (12:14)	Atrium
GSE115574 (78)	GPL570	59 (31:28)	Atrium
GSE31821	GPL570	6 (2:4)	Atrial appendage

### Identification of Robust Differentially Expressed Genes

**FIGURE 2:**
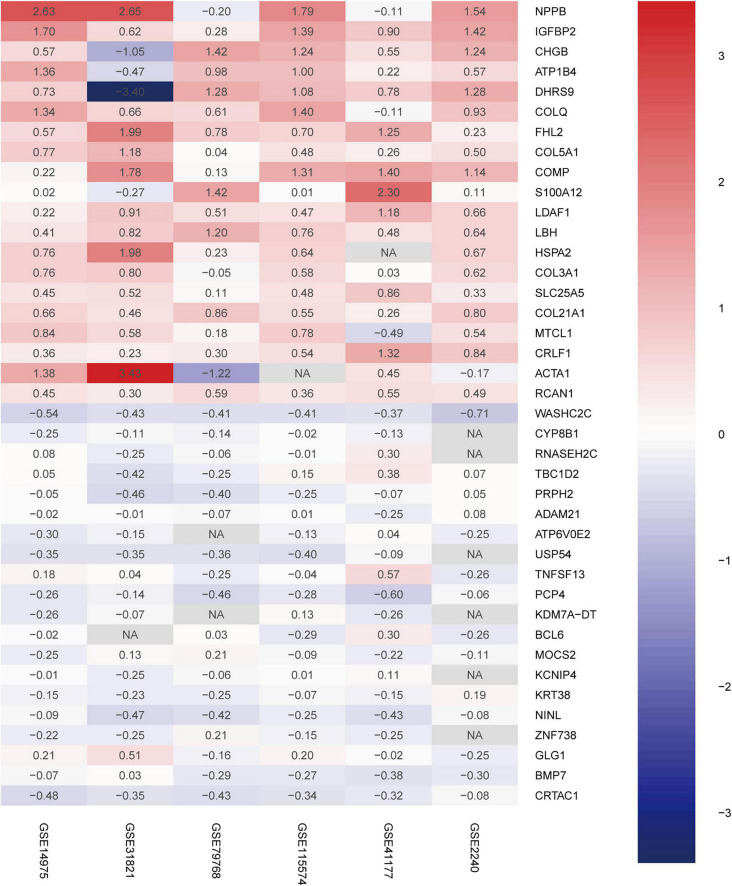
Robust DEGs identified by RRA analysis. Heatmap of the six datasets showing the top 20 upregulated and 20 downregulated DEGs. The horizontal axis indicates the gene name, and the vertical axis represents dataset name. Red indicates that the gene is upregulated in the AF patients compared with the SR individuals, and the blue represents downregulation. The number in a cell indicates the logFC of each gene in a dataset. DEG, differentially expressed gene; RRA, robust rank aggregation.

A total of 156 upregulated and 34 downregulated DEGs were identified using the RRA method (Supplementary Table 1). Using Phenolyzer,^10^ we confirmed identification of novel DEGs that were not reported previously (Supplementary Table 2). The top 20 upregulated and downregulated genes in AF are shown in a heatmap (Figure 2). Among these genes, caspase 3 (*CASP3*) (18), tumor necrosis factor (*TNF*) (19), and potassium voltage-gated channel subfamily H member 2 (*KCNH2*) had been previously characterized in AF (20). In contrast, *TRDN* antisense RNA 1 (*TRDN-AS1*) and *ADAM* metallopeptidase domain 21 (*ADAM21*) had not been previously associated with AF.

### Functional and Pathway Enrichment Analyses of Differentially Expressed Genes

**FIGURE 3:**
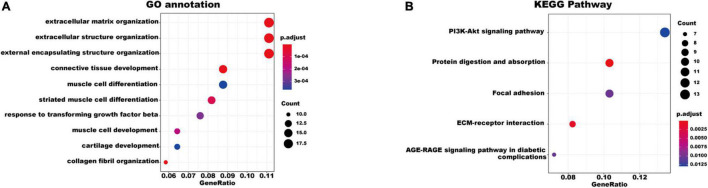
Gene Ontology and KEGG pathway enrichment of the robust DEGs in AF. **(A)** The enriched GO annotation terms. **(B)** The enriched KEGG pathway. GO, Gene Ontology; KEGG, Kyoto Encyclopedia of Genes and Genomes; DEG, differentially expressed gene.

**FIGURE 4:**
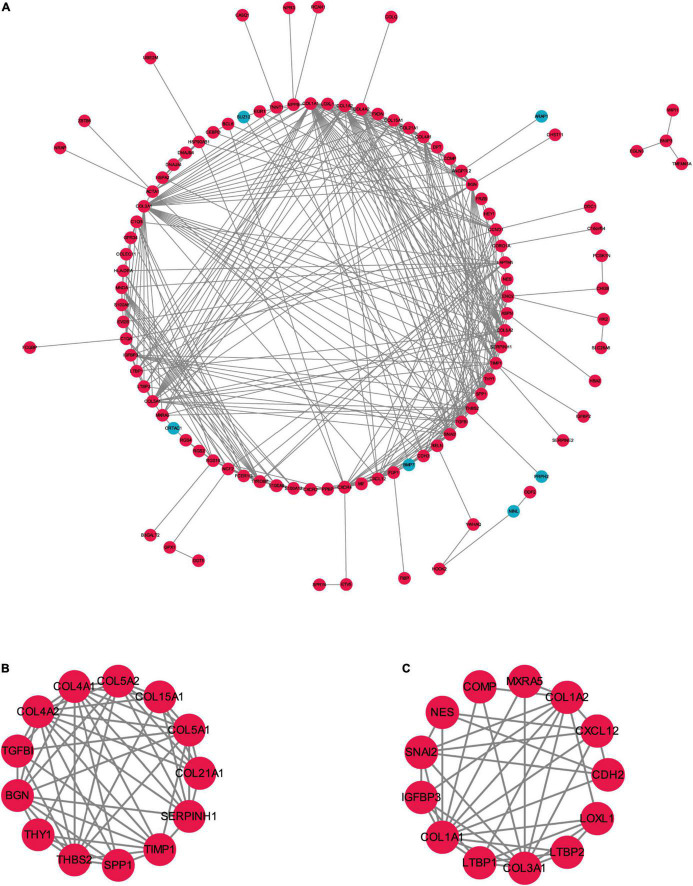
The PPI network of the robust DEGs. **(A)** PPI network of upregulated and downregulated significant genes. **(B,C)** The most significant two modules identified through MCODE in Cytoscape software. PPI, protein–protein interaction; DEG, differentially expressed gene. Red indicates upregulation of genes, and blue represents downregulation of genes in AF individuals.

To better understand the biological functions and characteristics of DEGs, we performed GO and KEGG analyses using “clusterProfiler” package. GO enrichment analysis showed that the DEGs were related to extracellular matrix formation, TGF-β response, and collagen fibril organization. In addition, KEGG pathway enrichment analysis indicated that pathways related to P13K-Akt signaling, protein dynamics, and ECM-receptor interaction were associated with AF (Figure 3). We then used the STRING database to construct a PPI network of these DEGs. The PPI network was visualized using Cytoscape 3.9.0 (Figure 4A). Then, the MCODE plugin was used to determine the top hub genes (Figures 4B,C). The Top 2 closely connected modules were identified. MCODE 1 included *BGN, COL21A1, SPP1, THY1, SERPINH1, COL15A1, TGFBI, COL5A1, TIMP1, COL5A2, COL4A2, COL4A1*, and *THBS2*. MCODE 2 included *COL3A1, SNAI2, CDH2, LTBP2, COL1A2, MXRA5, COL1A1, LOXL1, COMP, LTBP1, NES, CXCL12*, and *IGFBP3*.

### Weighted Gene Co-expression Network Analysis

**FIGURE 5:**
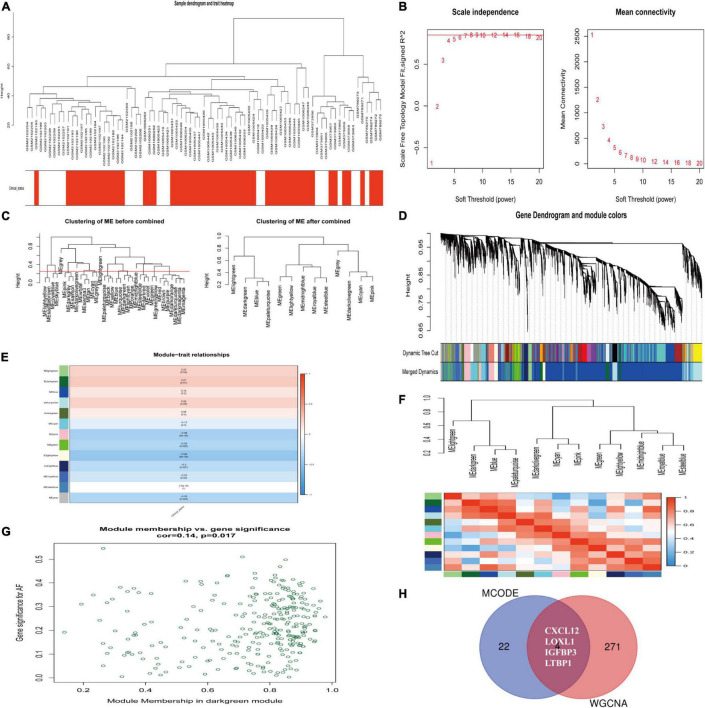
Identification of hub modules associated with AF by WGCNA. **(A)** Clustering dendrograms of genes from normalized six datasets. In the column of clinical status, red indicates AF, and white means the SR individuals. **(B)** The scale-free fit index (left) and the mean connectivity (right) for various soft-thresholding powers. **(C)** Clustering of module eigengenes. The cut height (red line) was 0.25. **(D)** Hierarchical cluster tree. **(E)** Heatmap showing the relationship between module eigengenes and clinical status. The numbers in cells mean the correlation coefficients and *p*-values. **(F)** Cluster analysis and heatmap of the genes in different modules. Red means a positive correlation, and blue indicates a negative correlation. **(G)** Scatter plot of module eigengenes in the dark-green module. **(H)** Venn diagram. The overlapped genes between WGCNA analysis and PPI analysis. AF, atrial fibrillation; SR, sinus rhythm. WGCNA, weighted gene coexpression network analysis; DEG, differentially expressed gene; TOM, topological overlap matrix; PPI, protein–protein interaction.

We constructed a weighted gene co-expression network based on genes with *p* value < 0.05 and logFCs > 0.25 from the ranked gene list to further investigate the significance of modules associated with AF. Thirteen modules were identified as important in AF by setting the soft thresholding power to 8 (scale-free R2 = 0.83) and cut height to 0.25 (Figures 5A–D). The correlations between module and clinical status are shown in a heatmap, and the dark-green module was most strongly associated with AF (Figures 5E,F). The dark-green module contained 275 genes, as shown in Figure 5G (correlation coefficient = 0.14, *p* = 0.017). A Venn diagram (Figure 5H) showed the genes that overlapped between the WGCNA and PPI analyses. The corresponding proteins in these hub genes interacted with each other closely, as determined using the PPI network constructed using the GeneMANIA (21) online tool^11^ (Supplementary Figure 2).

### Connectivity Map Analysis

**FIGURE 6:**
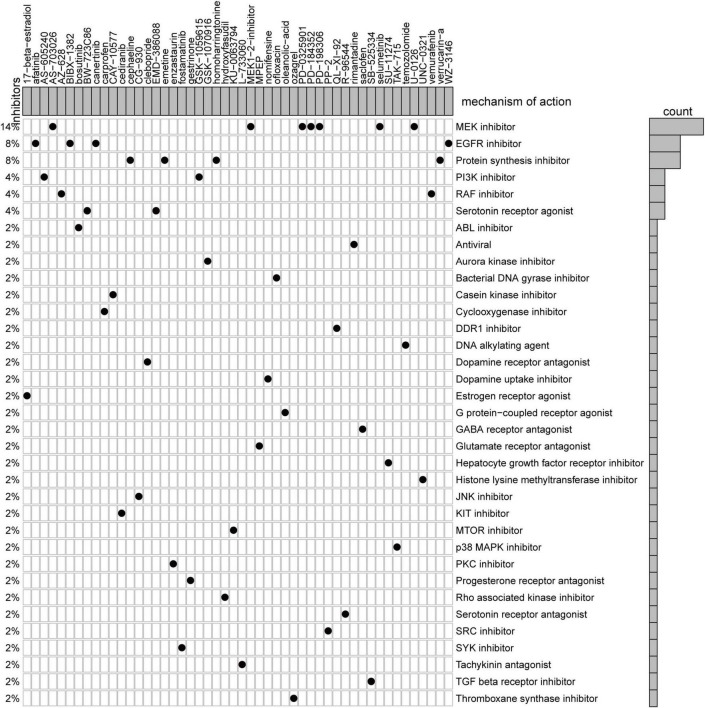
Heatmap showing each compound from the CMap that shares mechanisms of action (rows) and sorted by descending number of compounds with shared mechanisms of action.

We utilized CMap, a data-driven, systematic approach for investigating associations among genes, chemicals, and biological conditions, to identify potential compounds that targeted the AF gene signature (12). MoA analysis of the Top 50 compounds revealed 36 mechanisms of action shared by these compounds (Figure 6). Seven compounds (PubChemID: 44187362, 54483521, 10206158, 6918454, 9956637, 10127622, and 54539763) were mitogen-activated protein kinase (MEK) inhibitors. Four compounds (PubChemID: 10184653, 6918508, 156414, and 44607360) were epidermal growth factor receptor (EGFR) inhibitors.

### Integrative Analyses of *CXCL12*

**FIGURE 7:**
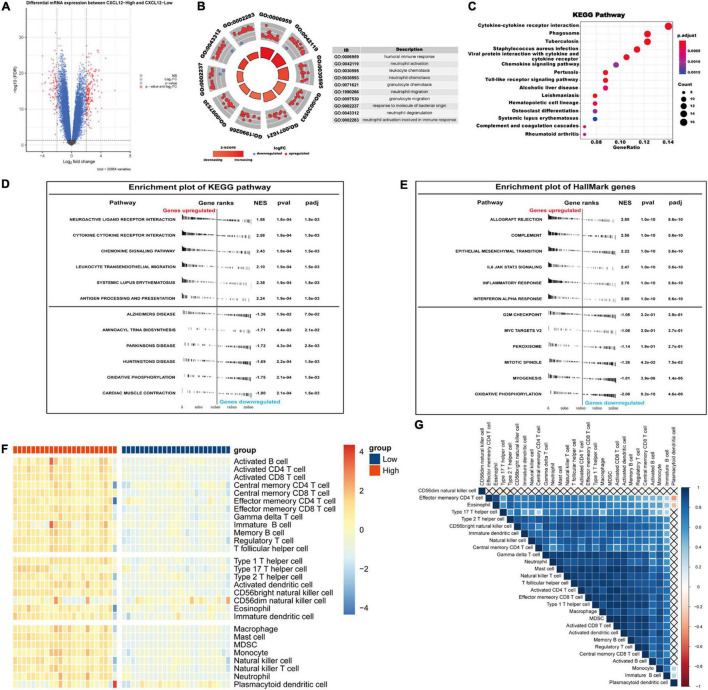
Gene Ontology/KEGG pathway enrichment of CXCL12-high and CXCL12-low group, GSEA and ssGSEA analysis of AF. **(A)** Volcano plot of DEGs between CXCL12-high and CXCL12-low group. **(B)** The top 10 enriched GO annotation terms. **(C)** The top 15 enriched KEGG pathway. **(D,E)** GSEA results in the context of gene sets for canonical pathways gene sets derived from the KEGG pathway database **(D)** and gene sets that contain genes annotated by the HallMark ontology term **(E)**. **(F)** Analyzing the types of immune cell infiltration in the CXCL12-high and CXCL12-low groups by ssGSEA. The color scale from blue to red indicates downregulation to upregulation. **(G)** Correlation heatmap among different immune cells. GSEA, Gene Set Enrichment Analysis; ssGSEA, single-sample Gene Set Enrichment Analysis.

We chose *CXCL12* for further analysis because it had the highest AUC. The expression level of *CXCL12* was determined using the quantile value for the AF cohort. DEGs between high *CXCL12* and low *CXCL12* groups were determined using the “limma” package in R software with the thresholds set to adjusted *p* value < 0.05 and LogFCs > 2 (Figure 7A). The results showed that 186 genes were up-regulated and 84 genes were down-regulated in the high *CXCL12* group. GO enrichment analysis was performed to further understand the influence of these DEGs on the biological features of AF. The top 10 enriched GO terms were mainly related to immune response and immune cell activity regulation (Figure 7B). In addition, KEGG pathway analysis suggested that the DEGs were enriched in cytokine-cytokine interaction, chemokine signaling pathway, toll-like receptor signaling pathway, and immune-related diseases (Figure 7C).

### Gene Set Enrichment Analysis, Single-Sample Gene Set Enrichment Analysis, and Immune Cell Infiltration Analysis

**FIGURE 8:**
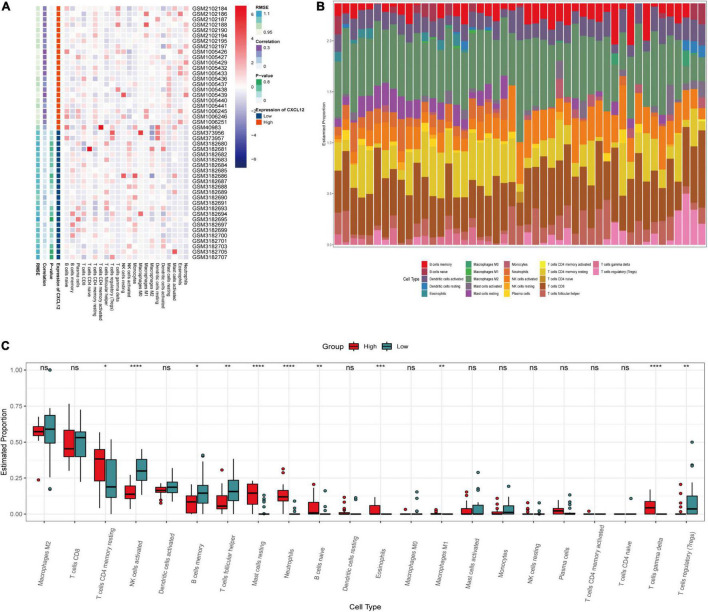
Immune infiltration analysis by CIBERSOT. **(A)** Heatplot showing the relative infiltration fraction of immune cells in CXCL12-high and CXCL12-low individuals. **(B)** Relative proportion of immune cell infiltration in CXCL12-high and CXCL12-low individuals. **(C)** Differences in immune cell infiltration between CXCL12-high and CXCL12-low individuals. *P < 0.05, **P < 0.01, *** *P* < 0.001, ****P < 0.0001, ns, no significance.

To investigate the biological functions associated with *CXCL12*, we performed GSEA analysis using the MSigDB hallmark gene sets and KEGG pathway gene sets in the high *CXCL12* and low *CXCL12* groups (Figures 7D,E). The results were consistent with those obtained from GO and KEGG analyses. Moreover, gene set variant analysis (GSVA) based on the ssGSEA algorithm was performed to evaluate the degree of enrichment of 28 immune cell types within each sample (Figures 7F,G). Different expression levels of *CXCL12* were associated with different immune cell infiltration profiles. We used the “CIBERSORT” algorithm to estimate the relative infiltration proportions of 22 immune cell types from AF samples, which produced similar results to those observed in the GSVA analysis. As indicated in Figures 8A–C, CD4 memory T cells, mast cells, neutrophils, and gamma delta (γδ) T cells showed greater infiltration in the high *CXCL12* group, and Treg cells showed lower levels of infiltration in the high *CXCL12* group. These results indicated that high *CXCL12* expression was associated with increased immune cell infiltration and might be associated with greater inflammatory activity. Use of different approaches confirmed the stability and repeatability of the findings, and suggested that *CXCL12* could be a marker to distinguish subsets of AF.

### Physicochemical Properties of *CXCL12*

**FIGURE 9:**
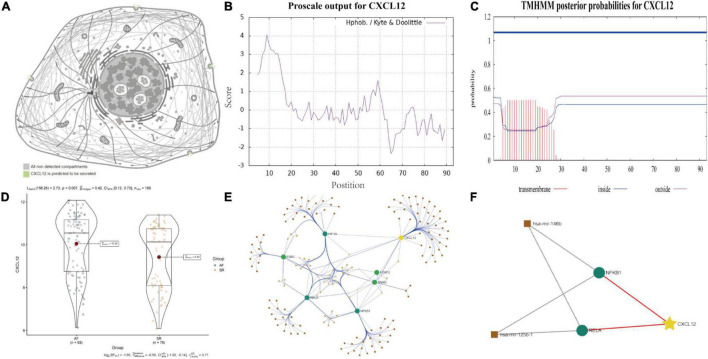
Biological features and regulation analysis of CXCL12 in AF. **(A)** The prediction model of the cellular location of CXCL12 protein. **(B)** The hydrophilcity/hydrophobicity analysis of CXCL12 protein by ProtScale. **(C)** The prediction of transmembrane helices of CXCL12 by TMHMM. **(D)** The different mRNA expression of *CXCL12* between AF and SR individuals. **(E)** TF-miRNA-mRNA regulation network for *CXCL12* in AF. **(F)** Sub regulation net for CXCL12 in AF based on DE-miRNAs identified by GSE28954 dataset.

ProtParam, ProtScale, and Protein Atlas analyses were used to interpret the physiochemical properties of CXCL12. The results showed that the CXCL12 protein consists 93 amino acids and has a half-life of 30 h in mammals. The amino acid composition of CXCL12 includes five negatively charged amino acid residues (Asp + Glu) and 16 positively charged amino acid residues (Arg + Lys). The theoretical isoelectric point is 9.92. The instability index of CXCL12 is estimated to be 22.75, which indicates that the protein is stable. In addition, the grand mean of the hydrophobic value is 0.082, which was consistent with the ProtScale analysis result showing that CXCL12 protein had similar numbers of hydrophobic and hydrophilic regions (Figure 9B). Analysis using Protein Atlas demonstrates that CXCL12 is a stable secretory protein (Figure 9A). Finally, CXCL12 does not include any transmembrane domains, as determined using the TMHMM server (Figure 9C).

### The Potential TF-miRNA-mRNA Regulatory Network for *CXCL12*

Studies have shown that miRNA and TF play essential roles in onset and progression of AF. We used the “limma” package in R software to identify DE-miRNAs with adjusted *p* value < 0.05 as the threshold (Supplementary Figure 3). We identified three up-regulated miRNAs and three down-regulated miRNAs. Among the DE-miRNAs, hsa-mir-146b and hsa-mir-125b were involved in regulation of TFs that mediated the expression of *CXCL12*, as shown in Figures 9D–F.

### Diagnostic Model

**FIGURE 10:**
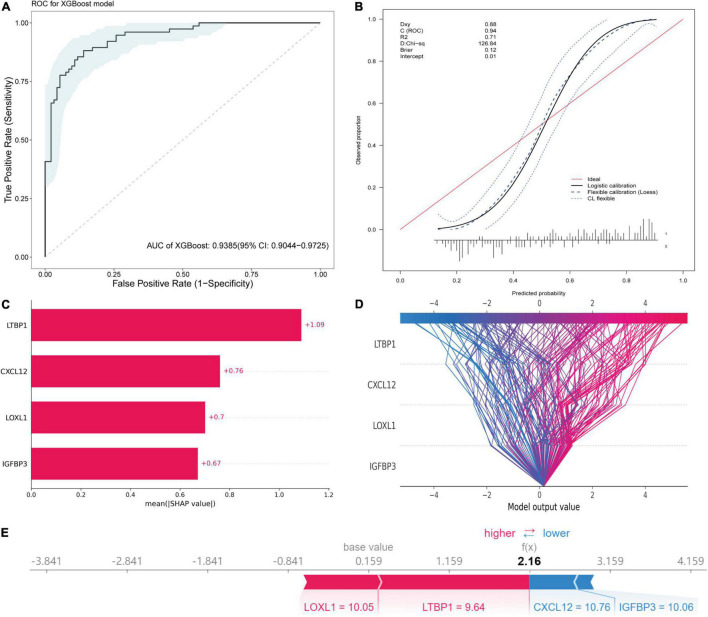
Diagnostic model construction, validation and visualization. **(A)** ROC plot of XGBoost algorithm-based diagnostic model. **(B)** The calibration curve of the diagnostic model. **(C)** Importance plot of the diagnostic model. **(D)** The decision plot showing global interpretability of the model. **(E)** The force plot showing example explanation on an individual case.

An XGBoost classification model was used to construct a diagnostic model, whose parameters were listed in Supplementary Table 4. All four hub genes were selected as variables. The model was internally validated using the bootstrap method with 1,000 resampling iterations. The model resulted in an AUC of 0.9385 (95% CI: 0.9044–0.9725; Figure 10A). The calibration plot is shown in Figure 10B. The Brier score was 0.12, which verified the reliability of the model.

### Model Interpretation Using the Sharpley Additive exPlanations

The variance importance plot, decision plot, and force plot from the original set are shown in Figures 10C–E. For each prediction, the SHAP value was positively associated with risk of AF. The features were ordered according to the importance scores of each value in this model (Figure 10C). Higher values of *CXCL12*, *LOXL1*, and *IGFBP3* (than the average) were strongly associated with diagnosis of AF, and lower values of *LTBP1* (than the average) were associated with diagnosis of AF (Supplementary Figure 4). The decision plot (Figure 10D) showed global interpretability of the model, whereas the force plot showed local interpretability (explanation of an individual case) (Figure 10E).

## Discussion

The mechanism of AF is complex and heterogeneous (22, 23). Although some actionable and reversible precipitants are identified, like hyperthyroidism (24), endurance sport (25), alcohol consumption (26), sleep disordered breathing (27), channelopathies (28), the etiology and pathogenesis of AF are still waiting to be clarified.

**FIGURE 11:**
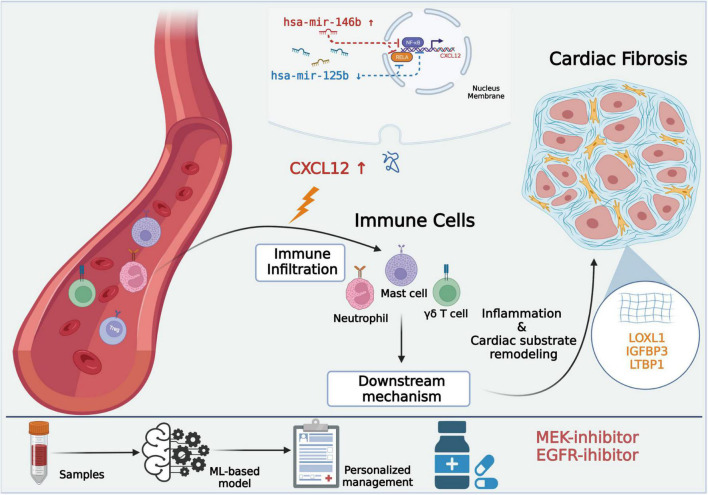
Central illustration of the key findings. *CXCL12* may play as an upstream mediator and its TF-miRNA-mRNA regulation network was shown in the graph. *LOXL1, IGFBP3, LTBP1* may impose important effect on atrial fibrosis, which was the pathogenetic cornerstone of AF. The ML-based model could be used in personalized diagnosis and management. MEK inhibitor and EGFR inhibitor may be potential non-channel drugs for atrial fibrillation. AF, atrial fibrillation. ML, machine learning. MEK, mitogen-activated protein kinase. EGFR, epidermal growth factor receptor (Created with BioRender.com).

In the current study, we included datasets derived from atrium or sleeve of pulmonary vein tissues to minimize influences from peripheral blood on the results. We identified four hub genes that were closely interacted. Besides, potential small molecule compounds, underlying molecular regulatory mechanism of AF were investigated by integrating bioinformatic tools. Then, we constructed a reliable diagnostic model using the identified hub genes, which would aid in personalized management (Figure 11).

### CXCL12 May Be an Upstream Mediator of Atrial Fibrillation and a Biomarker for Identification of Atrial Fibrillation Subsets

Previous study showed that CXCL12 was associated with anabatic atrial inflammation and fibrosis (29) and its serum concentration varied among sinus rhythm, paroxysmal AF and persistent AF individuals (30). Besides, the increased CXCL12 in plasma was associated with AF progression (31, 32). However, the role of CXCL12 in the local inflammatory microenvironment regulation and its regulatory network in AF was not fully revealed. Our study indicated that CXCL12 might perform as an important regulator of inflammation in AF by increasing the infiltration of mast cells, neutrophils, and γδ T cells, and reducing infiltration of regulatory T cells. Studies have shown that neutrophils (33) were the main source of reactive oxygen species (ROS) and myeloperoxidase (MPO), which are highly associated with fibrosis in AF. One study (34) suggested that atrial fibrosis and collagen deposition could be reversed by a mast cell stabilizer and a PDGF-A blocker, which indicated that mast cells might be therapeutic targets to control atrial fibrosis. Dumitriu et al. showed that levels of anti-inflammatory Tregs were significantly reduced in AF (35), and He et al. reported that a higher Th17/Treg ratio in serum predicted onset of post-operative AF (36). Recently, Zhang et al. also suggested that oral administration of *B. fragilis* attenuated the inflammatory response by increasing infiltration of Treg cells, thereby preventing age-related AF (37). The present study showed that *CXCL12* was associated with regulation of Treg infiltration, which represented a novel mechanism of Treg regulation in AF.

Collectively, the immune infiltration analysis in the current study suggested that CXCL12 might play a crucial role in induction of inflammation and immune cell infiltration in AF. Cytokine antagonists or CXCR4 inhibitors (29) might be effective therapeutic agents. The present study also suggested that *CXCL12* expression might be a marker for determination of AF subsets for making actionable personalized treatment plans. Besides, in current study, we found that the CXCL12 expression could be regulated by a TF-miRNA-mRNA network. Previous study reported the role of miR-146b-5p in atrial fibrosis in AF by repressing TIMP-4 (38, 39). Our result showed that miR-146b-5p might participate in local inflammation regulation in AF by negatively regulating NF-κB. The downregulation of miR-125b was reported in valvular AF patients (40). However, the role of miR-125b in the NF-κB pathway regulation varied in different diseases background (41, 42). Further studies need to confirm its role in AF setting.

### The Lysyl Oxidase/Related Lysyl Oxidase-Like Family, LTBP1, and IGFBP3 Might Contribute to Atrium Substrate Remodeling

Lysyl oxidase (LOX) and related LOX-like (LOXL) isoforms play vital roles in remodeling of the extracellular matrix (ECM) (43). LOXL1 is a member in LOX family with highly similar catalytic domains as LOX. It has been characterized in diseases such as exfoliation syndrome (44), cardiac hypertrophy (45), and endothelial dysfunction, and is thought to be essential for elastic fiber homeostasis (46). Recent studies have shown that LOX/LOXL inhibitors modulated fibrotic atrial remodeling (47, 48). Interestingly, LOXL2, another member of the LOX family, was highly expressed in patients with permanent atrial fibrillation (49). Further characterization of the role of LOX-family proteins in AF and development of inhibitors with better specificity and reduced side effects (50) may result in better therapeutic performance.

LTBP1 is the major source of TGF-β (51) in the ECM. LTBP1 forms a disulfide linked complex with the TGF-β propeptide in the endoplasmic reticulum prior to secretion, and promotes TGF-β activation (52). Stacy et al. reported that activation of LTBP1 was associated with regional atrial fibrosis and vulnerability to AF following myocardial infarction (53).

IGFBP3, which is the main binding target of IGF-1, plays an important role in regulating the activity and transport of IGF-1, and was reported to be independently associated with AF in the elderly population in the System-IGF-1 Pathway and Alzheimer’s Disease Clinical Trial (SIGAL) (54). Busch et al. suggested that low IGF-1/IGFBP-3 ratios were associated with a higher prevalence of AF in the Study of Health in Pomerania (SHIP) (55). Our study confirmed that transcript levels of *IGFBP-3* were associated with AF and that *IGFBP-3* may be a biomarker of AF.

### Mitogen-Activated Protein Kinase/Epidermal Growth Factor Receptor Inhibitors Are Promising Pharmacologic Interference Targets for Atrial fibrillation

Treatment options for AF exhibit limited efficacy. Pharmacotherapy identifying interventions which target atrial cardiomyopathy evolution (56) might postpone or even prevent the development of AF (57) and it deserves high priority. We found that MEK inhibitors and EGFR inhibitors may be promising agents for treatment of AF.

Activation of the MEK/ERK-MAPK cascade by the Ang-II signaling pathway has been previously associated with AF (58, 59). Specific inhibition of MEK and ERK with PD98059 or U0126 during AF may prevent fibrous tissue formation (60). It was serendipity that some studies had indicated that patients with melanoma were at reduced risk for development of AF when treated with BRAF and MEK inhibitors compared to patients treated with BRAF inhibitor monotherapy (61).

Epidermal growth factor receptor inhibitors have typically been used for treatment of cancer. However, increasing evidence has supported use of EGFR inhibitors to treat cardiovascular diseases based on the ability of these inhibitors to regulate EGFR-AT1R crosstalk (62). Recent studies of EGFR-AT1R crosstalk have focused on cardiac hypertrophy (63), smooth vascular cell dysfunction (64), vascular remodeling (65), and ECM formation (66). The relationship between EGFR-AT1R crosstalk and AF has not been elucidated. Although administration of ibrutinib (a Bruton tyrosine kinase inhibitor) was shown to increase the risk for development of AF, the pro-arrhythmic effects of ibrutinib resulted from inhibition of C-terminal Src kinase but not inhibition of tyrosine kinase (67).

A previous study showed an association between genetic variants in the EFGR gene locus and AF progression (68). A subsequent study showed that EGF and heparin-binding EGF-like growth factor levels in patients with AF were significantly higher than those in control individuals (69). Further investigation is needed to characterize the relationship between EGFR and AF.

### Diagnostic Model for Use in Clinical Decision Making

In the present study, we developed and validated an interpretable ML-based diagnostic tool for prediction of AF. Our findings indicated that the model exhibited excellent discriminatory performance, with an average AUC of 0.9385. Using the SHAP method, we visualized the model to help users understand the complex integration model. Since the included genes were closely related to the mechanism of AF, we anticipate that this model will have clinical applicability for prediction of progression and recurrence of AF.

Collectively, it is unlikely that there will be an “one-size-fit-for-all” option for AF management, it will be essential to identify the subset of AF patients who are most likely to benefit from a given therapy. Enormous progress in radiology (70–72) and multiomics study (73) has changed our view about the management of AF, and we are expecting to conduct more integrative, precise and personalized therapeutic practice for AF in the future.

### Limitations

The current study only included bioinformatics analyses. Future *in vitro* and *in vivo* studies will be needed to explore the molecular mechanisms and pathways identified in this study. The current work was based on transcriptomic data, in which mutational variance was not investigated. The inflammatory mechanisms among AF patients might vary. Considering that *CXCL12* is an important pro-inflammatory cytokine-related gene, so we conducted non-negative matrix factorization clustering analysis based on cytokine-related genes derived from Immport database^12^ and found that included samples could be divided into four cytokine-related subgroups which showed distinctive expression pattern (Supplementary Figure 6 and Supplementary Table 6). However, owing to the lack of detailed clinical data of the included patients, further investigations are required to identify the clinical significance and feasibility of this framework. Additionally, external validation of the ML-based model generated in this study is required to verify its robustness and efficacy in other data sets.

## Conclusion

In this study, we identified four key genes involved in the pathogenesis of AF, and identified potential therapeutic targets for treatment of AF. The biological features and regulatory mechanisms of *CXCL12* in AF were comprehensively investigated using integrative bioinformatics tools. The results indicated that *CXCL12* might be a potential marker to distinguish AF subsets, and showed that it could be an important intermediate between the local inflammatory microenvironment and atrial fibrosis. A reliable ML-based diagnostic model was constructed that is suitable for evaluation of AF progression and recurrence. Our work provided novel insights into AF and generated an effective tool that could be used in clinical practice.

## Data Availability Statement

The data presented in this study are available in the GEO database under accession numbers GSE2240, GSE14975, GSE41177, GSE79768, GSE115574, GSE31821, and GSE28954 (https://www.ncbi.nlm.nih.gov/geo/query/acc.cgi?acc).

## Author Contributions

LY and YC conceived and performed the study. WH reviewed and edited the manuscript. All authors read and approved the manuscript.

## Conflict of Interest

The authors declare that the research was conducted in the absence of any commercial or financial relationships that could be construed as a potential conflict of interest.

## Publisher’s Note

All claims expressed in this article are solely those of the authors and do not necessarily represent those of their affiliated organizations, or those of the publisher, the editors and the reviewers. Any product that may be evaluated in this article, or claim that may be made by its manufacturer, is not guaranteed or endorsed by the publisher.

## Footnotes

1
https://www.ncbi.nlm.nih.gov/geo/

2
http://geneontology.org/docs/go-citation-policy/

3
https://www.genome.jp/kegg/kegg1.html

4
https://string-db.org/cgi/input.pl

5
https://clue.io/

6
https://www.mirnet.ca/miRNet/home.xhtml

7
https://www.proteinatlas.org/

8
https://web.expasy.org

9
http://www.cbs.dtu.dk/services/TMHMM

10
https://phenolyzer.wglab.org/

11
http://www.genemania.org

12
https://www.immport.org/resources
