# Supplementary material for: Hub Genes Identification, Small Molecule Compounds Prediction for Atrial Fibrillation and Diagnostic Model Construction Based on XGBoost Algorithm
Source: Front Cardiovasc Med. 2022 Jul 14;9:920399. doi: 10.3389/fcvm.2022.920399 (PMC9329605; doi:10.3389/fcvm.2022.920399)
Supplement: Supplementary file 1 [file Data_Sheet_1.docx]

Supplementary Material

# Supplementary Figures


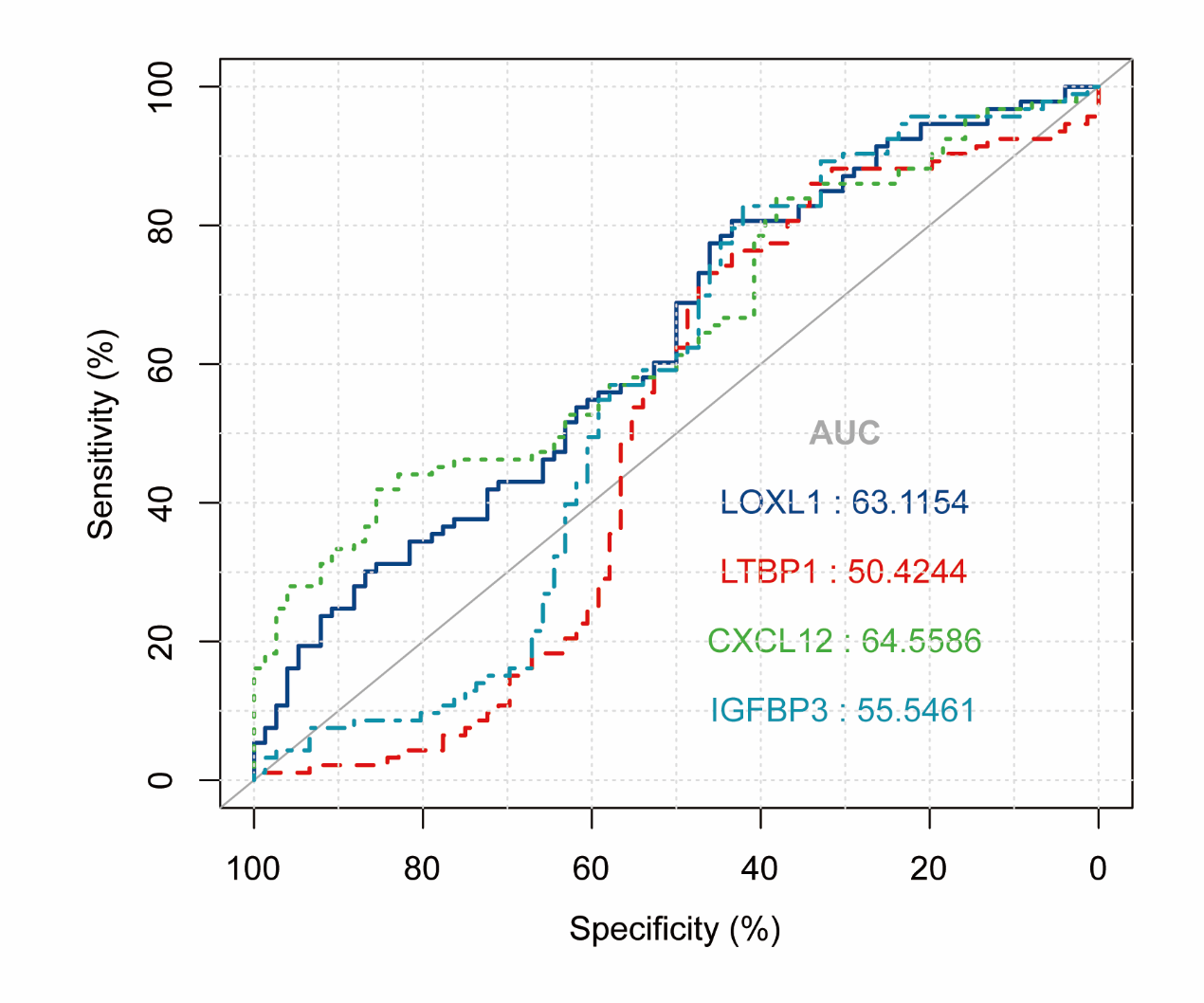


## Supplementary Figure 1. Receive operative curves of four hub genes.


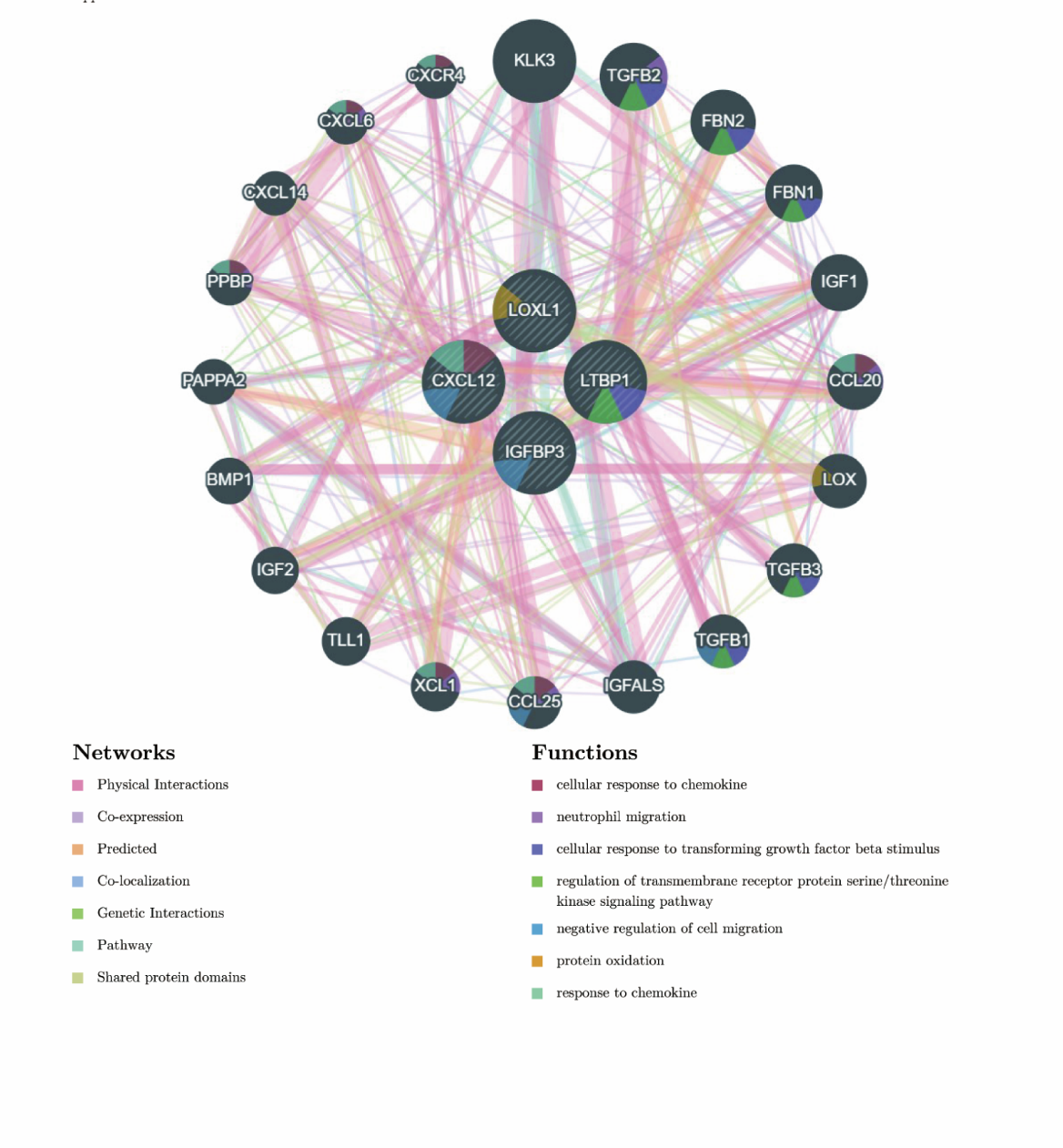


## Supplementary Figure 2. Interaction among the hub genes using GeneMANIA methods.


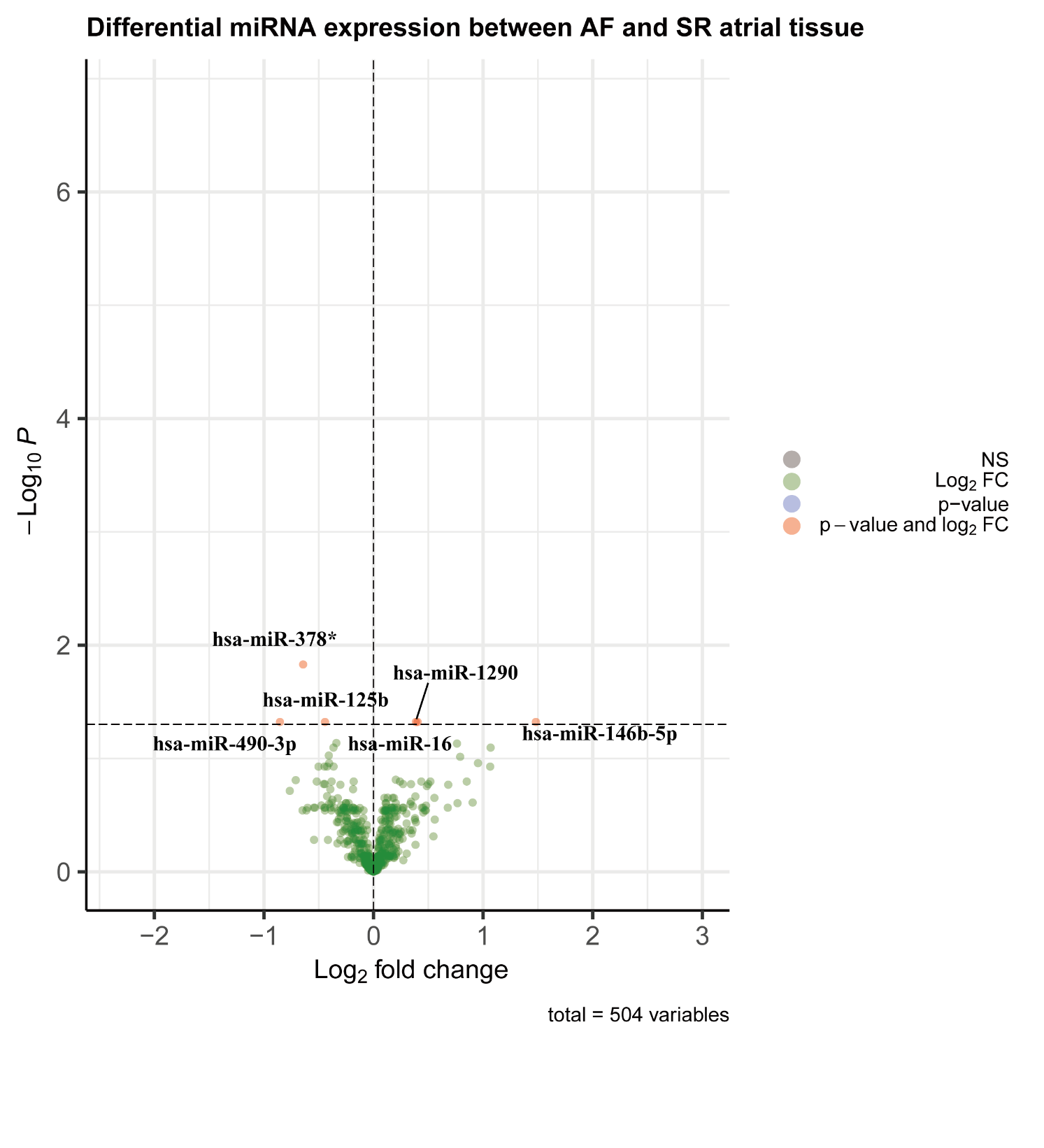


## Supplementary Figure 3. Volcano plot of DE-miRNA between AF and SR based on dataset GSE28954.


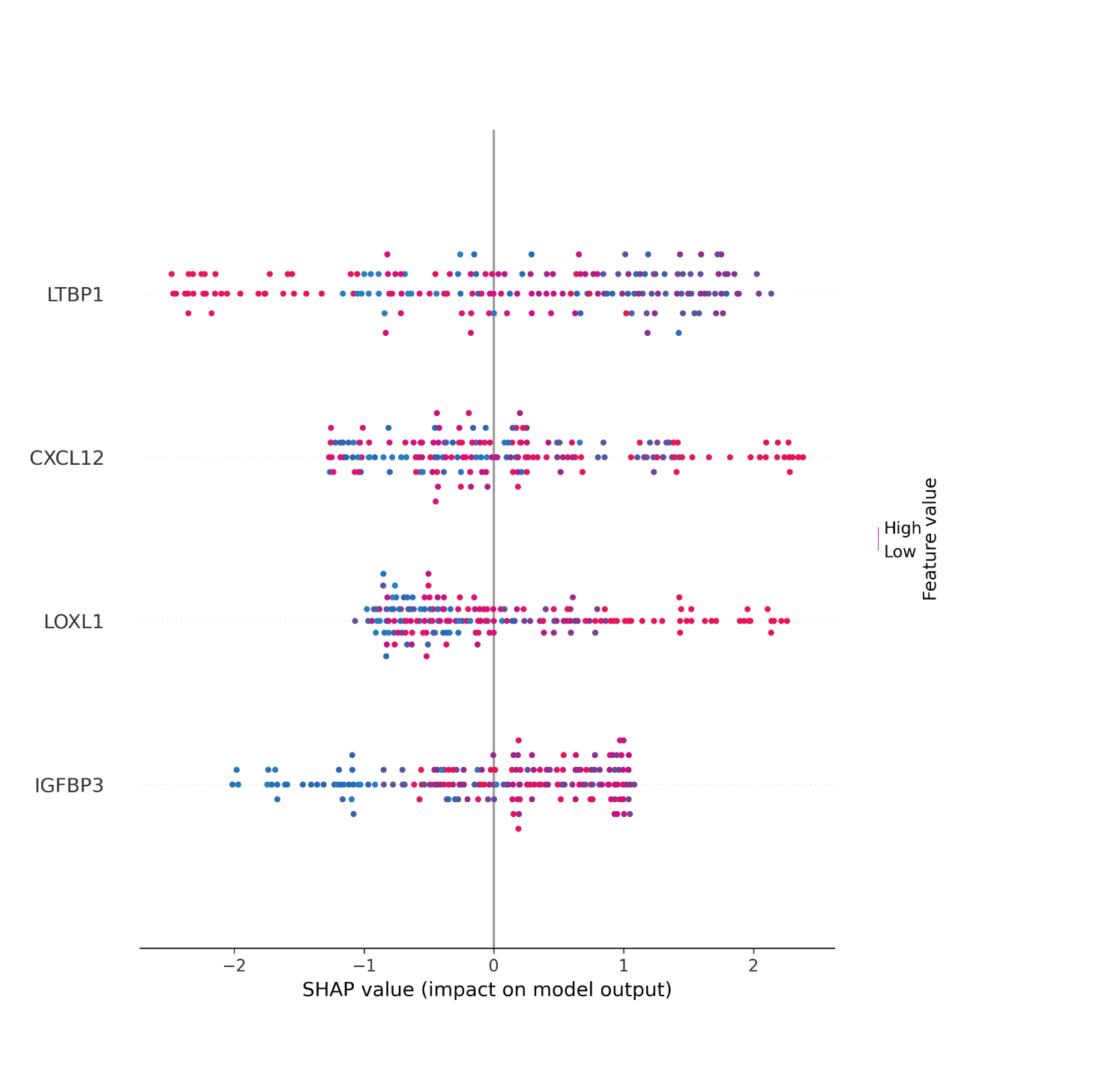


## Supplementary Figure 4. Global feature importance by SHAP for the hub genes.

##
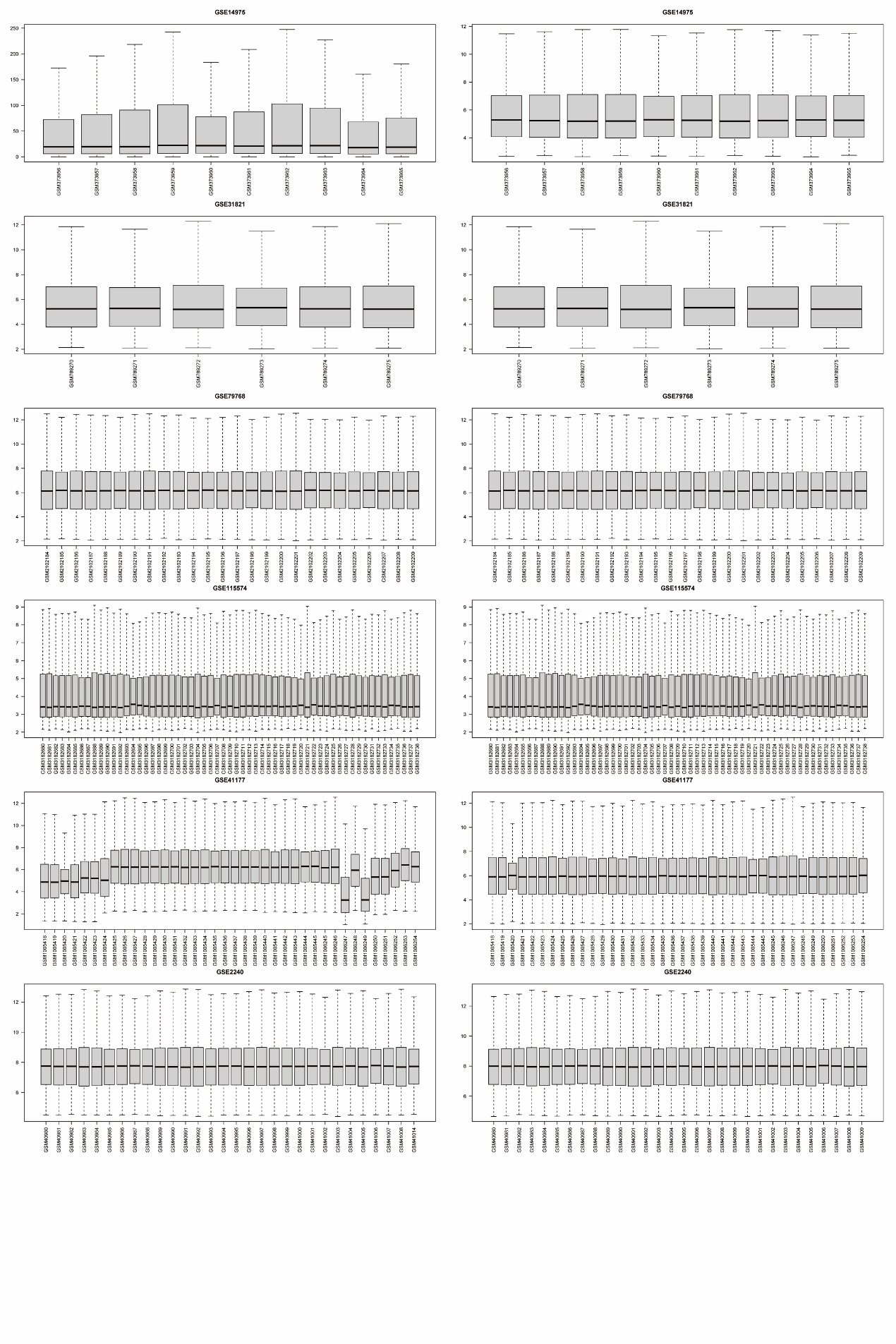


## Supplementary Figure 5. Boxplots of six gene sets before and after normalization.

## Supplementary Figure 6. (A) Heat map showed the expressions of cytokine-related genes in four clusters. (B) NMF consensus clustering for the k value was 4. (C)NMF distributions when rank = 2-10. (D) Venn diagram showed the genes that overlapped among different clusters.


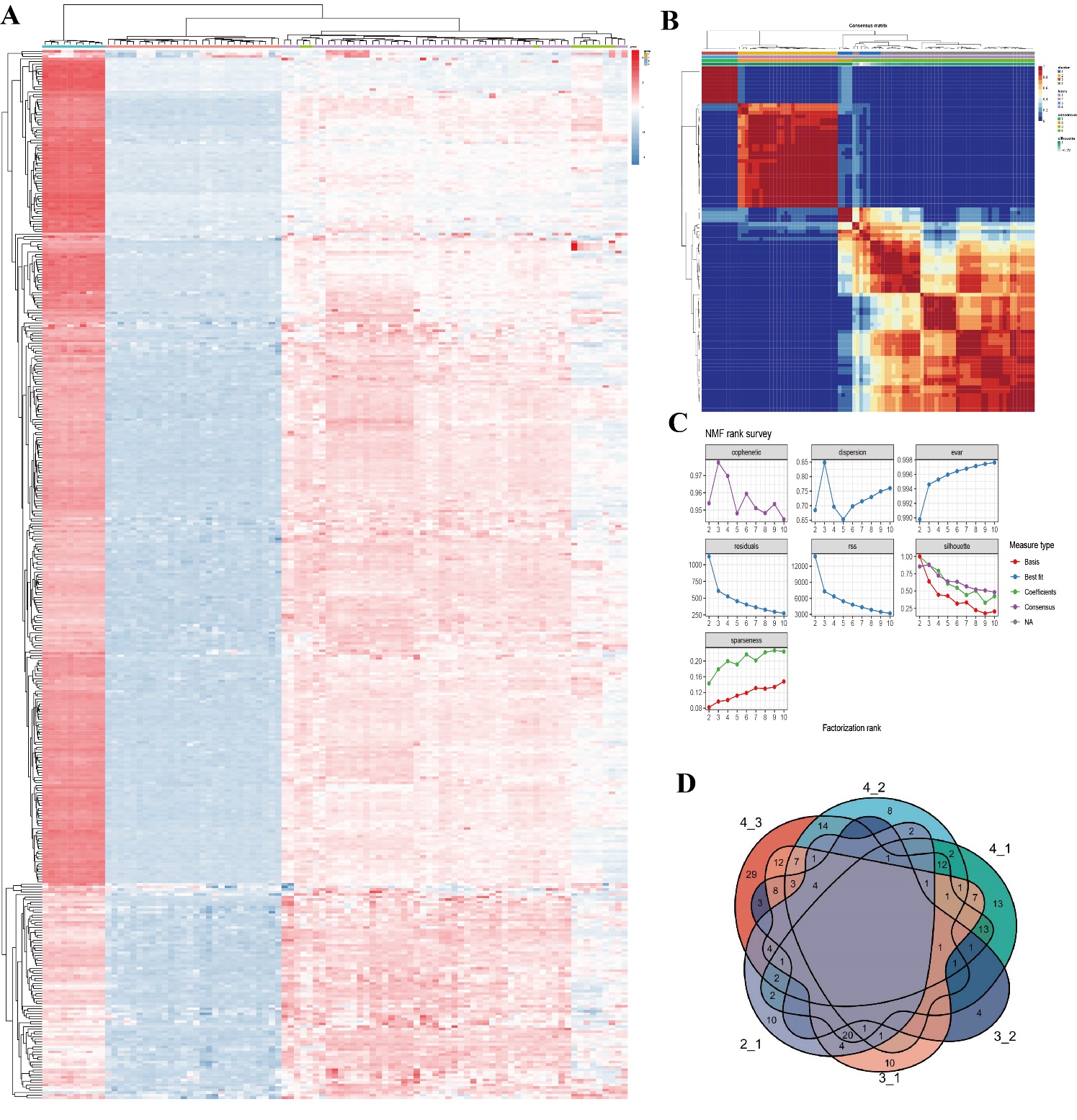


# Supplementary Tables

## Supplementary Table 1. DEGs identified by RRA method.

|  | **GSE14975** | **GSE31821** | **GSE79768** | **GSE115574** | **GSE41177** | **GSE2240** |
| --- | --- | --- | --- | --- | --- | --- |
| **NPPB** | 2.629724478 | 2.648367245 | -0.195813259 | 1.793478211 | -0.110105225 | 1.540846469 |
| **IGFBP2** | 1.700660988 | 0.619487768 | 0.281171534 | 1.387019527 | 0.8990958 | 1.420726037 |
| **CHGB** | 0.574769166 | -1.047987404 | 1.418161882 | 1.244984618 | 0.55199306 | 1.240710605 |
| **ATP1B4** | 1.363807098 | -0.47236998 | 0.978339273 | 0.996372694 | 0.224677803 | 0.56785744 |
| **DHRS9** | 0.72583641 | -3.397812522 | 1.280909619 | 1.078656947 | 0.775008192 | 1.283521391 |
| **COLQ** | 1.339220304 | 0.66483572 | 0.608134679 | 1.401442724 | -0.110615945 | 0.92912267 |
| **FHL2** | 0.574350779 | 1.994184394 | 0.781809036 | 0.699617978 | 1.254663342 | 0.226762288 |
| **COL5A1** | 0.772388955 | 1.177720238 | 0.043566582 | 0.477512103 | 0.261885102 | 0.503222785 |
| **COMP** | 0.21945791 | 1.782426372 | 0.130366818 | 1.308015359 | 1.395330518 | 1.142101448 |
| **S100A12** | 0.018017294 | -0.27455521 | 1.422056128 | 0.00987528 | 2.297543982 | 0.114661334 |
| **LDAF1** | 0.224360945 | 0.905116031 | 0.511259051 | 0.469204148 | 1.176369906 | 0.663804851 |
| **LBH** | 0.414673993 | 0.817462942 | 1.204156682 | 0.760217722 | 0.482408993 | 0.639161019 |
| **HSPA2** | 0.760916473 | 1.977697418 | 0.228900715 | 0.637958334 | NA | 0.666996738 |
| **COL3A1** | 0.764344467 | 0.798210908 | -0.053433983 | 0.575877089 | 0.032810389 | 0.624978363 |
| **SLC25A5** | 0.449146187 | 0.515822331 | 0.111272246 | 0.478870143 | 0.862402314 | 0.332644132 |
| **COL21A1** | 0.660402764 | 0.458647367 | 0.855660617 | 0.54853092 | 0.262030911 | 0.802037278 |
| **MTCL1** | 0.841376103 | 0.576036115 | 0.184141877 | 0.775175433 | -0.491116716 | 0.540057587 |
| **CRLF1** | 0.358393169 | 0.231966545 | 0.302641878 | 0.543452594 | 1.318295943 | 0.843604154 |
| **ACTA1** | 1.375741334 | 3.432588962 | -1.220156892 | NA | 0.448349095 | -0.172572345 |
| **RCAN1** | 0.446154569 | 0.304731909 | 0.588576457 | 0.364847793 | 0.545435686 | 0.490188679 |
| **CXCR2** | 0.081625613 | -0.309094898 | 1.254836644 | -0.053361618 | 2.308197802 | 0.015497466 |
| **FGF1** | 0.111703174 | 0.933757216 | 0.565990474 | 0.323163066 | NA | 0.306778932 |
| **RELN** | 0.733172121 | -0.945128352 | 1.222238887 | 1.134024436 | -0.755021278 | 0.383353131 |
| **COL4A2** | 0.577080397 | 1.070701543 | 0.231433874 | 0.322601239 | 0.530837117 | 0.488256318 |
| **PHLDA1** | 0.598363103 | -0.056360749 | 0.242102521 | 0.60732774 | NA | 0.470366837 |
| **MAP3K7CL** | 0.581259297 | 2.178659872 | 0.08268573 | 0.317217795 | -0.096778935 | 0.360363126 |
| **COL1A2** | 0.586712284 | 0.696575344 | -0.072330162 | 0.574694764 | 0.331377599 | 0.490885816 |
| **TRDN-AS1** | -0.907093399 | 0.300841119 | 1.636580137 | 1.03249011 | 0.248807209 | NA |
| **CES2** | 0.355216029 | 0.978104474 | 0.19008586 | 0.472123819 | 0.236674494 | 0.298103194 |
| **ASPN** | 0.191076405 | 0.953274827 | 0.737851554 | 0.458336282 | 0.188993233 | 0.673961198 |
| **RNF216** | 0.679648426 | -0.06067701 | 0.167525889 | 0.502934972 | 0.212373819 | 0.422582881 |
| **PLEKHO1** | 0.621562861 | 0.620860097 | 0.22220537 | 0.477004969 | 0.398448428 | 0.304646433 |
| **DGKI** | 0.981472602 | -0.207426837 | 0.183166895 | 0.908035558 | -0.373046137 | 0.412549176 |
| **CXCR4** | 0.451501161 | 1.048869181 | 0.704780199 | 0.29218694 | 1.202635876 | 0.212887554 |
| **MNDA** | 0.207658823 | -0.475957882 | 1.16743064 | 0.111946874 | 1.534360925 | 0.02160945 |
| **MXRA5** | -0.563932753 | 1.737084134 | -0.142135218 | 1.06108916 | -0.254140148 | 0.767538464 |
| **DIRAS3** | 0.786042694 | 0.934550089 | 0.310191884 | 0.923754188 | 0.496186016 | 1.045242017 |
| **DPT** | 0.403501278 | 1.086198882 | 0.102448718 | 0.477821384 | 1.073432758 | -0.141600124 |
| **ANGPTL2** | 0.538973466 | 0.43070711 | 0.139513526 | 0.749548349 | 0.407694843 | 0.585415011 |
| **ALPK3** | 0.460600502 | 0.97733095 | 0.032012921 | 0.462380578 | -0.475846683 | 0.391204177 |
| **LOC101928304** | 1.016386785 | -0.904000093 | 0.692940572 | 0.759662898 | 0.206279593 | NA |
| **LTBP2** | 0.427391844 | 0.157786118 | -0.055836834 | 0.814772351 | 0.355291959 | 0.727266916 |
| **PXDN** | 0.598495041 | 0.576983021 | -0.264700081 | 0.418654946 | 0.25469725 | 0.563815865 |
| **RPL3L** | 0.091776401 | 0.339605824 | 0.64557003 | 0.949733479 | 0.668575423 | 0.705774809 |
| **SPRY4** | 0.957666463 | 0.353116228 | -0.164959565 | 0.472382498 | 0.091759254 | 0.369556578 |
| **NES** | 0.530390229 | 0.403524192 | -0.181012055 | 0.633883551 | 0.055049994 | 0.360447807 |
| **THBS2** | 0.683082171 | 0.543573854 | 0.167054732 | 0.499733638 | 0.305648307 | 0.357840097 |
| **HOOK2** | 0.288098751 | 1.464838159 | 0.141714754 | 0.41776098 | 0.16492185 | 0.352359645 |
| **IGFBP3** | 0.938411397 | 0.94070882 | 0.289272278 | 0.43585295 | 1.005023977 | -0.065403832 |
| **CLC** | -0.0067538 | -0.330737391 | 0.977997439 | -0.056493994 | 1.366370604 | 0.08611156 |
| **BGN** | 0.467357481 | 0.641750257 | NA | 0.407203308 | 0.50996691 | 0.348086189 |
| **PCSK1N** | 0.583411396 | -0.540634386 | 0.119794101 | 0.389855965 | -0.077787784 | 0.466109473 |
| **HSP90AB1** | 0.187934515 | 1.660564614 | 0.469482213 | 0.420084776 | 0.88376926 | -0.123931582 |
| **P2RX5** | 0.904971771 | 0.586667457 | -0.13216019 | 0.539539228 | 0.219540481 | 0.343169642 |
| **QPCT** | 0.082045708 | 1.878252838 | 0.468777842 | 0.255307547 | 0.424980489 | 0.390220613 |
| **POPDC3** | 0.105697394 | -0.240148443 | 0.636161431 | 0.480650756 | 0.613080015 | 0.426859878 |
| **LOC101927069** | 0.445446284 | 1.585692446 | 0.283972287 | 0.704166235 | 0.375670402 | NA |
| **NRAP** | 0.152777162 | 0.765471108 | 1.04984074 | 0.182505066 | 1.349522549 | -0.388791675 |
| **COLEC11** | 0.818675981 | 1.664546048 | -0.379293419 | 0.073442392 | 0.191865246 | -0.421411161 |
| **COL4A1** | 0.497400953 | 0.816400656 | 0.151960987 | 0.21900384 | 0.653385184 | 0.508109969 |
| **TIMP1** | 1.076410172 | 0.715741538 | -0.495064627 | 0.377342984 | 1.034405799 | 0.155803336 |
| **TNNT1** | 0.223534048 | 1.042106899 | -0.159749858 | 0.65920679 | 0.114441808 | 0.702382397 |
| **KCNJ4** | 0.27313874 | 1.10185159 | 0.088298181 | 0.373158436 | 0.118906049 | 0.364418792 |
| **HK2** | 0.324342279 | -0.226692906 | 0.188371064 | 0.68884014 | -0.112552183 | 0.639940288 |
| **ALYREF** | 0.234464561 | 0.785380935 | 0.684049128 | 0.072959249 | 1.08022946 | NA |
| **UNC5B-AS1** | 0.747980853 | -0.215361845 | 0.450807253 | 0.858605553 | 0.545068472 | NA |
| **WIPI1** | 0.262325266 | 0.696802454 | NA | 0.406313441 | 0.090570364 | 0.419022101 |
| **RGS4** | 0.9836533 | 0.23927247 | 0.243660648 | 0.35419106 | 0.084224391 | 0.357885247 |
| **MPV17** | 0.373177402 | 0.435142934 | 0.190619986 | 0.323175562 | 0.527819664 | 0.301675271 |
| **PRICKLE1** | 0.550008987 | 1.149662537 | 0.239173224 | 0.351170079 | -0.021915765 | NA |
| **COL1A1** | 0.406531063 | 0.570334421 | 0.222828646 | 0.409439037 | NA | 0.554976018 |
| **DPYSL4** | 0.107169681 | 0.44426514 | 0.659536861 | 0.345252132 | 0.329500096 | 0.577840488 |
| **YWHAQ** | 0.392756746 | 0.54686226 | 0.127862916 | 0.424683991 | 0.699303732 | 0.246500857 |
| **ATP13A3** | 0.822530092 | NA | 0.557484598 | 0.544920115 | -0.135321152 | 0.060152813 |
| **ETV5** | 0.406042212 | -0.137422672 | -0.013960742 | 0.33337995 | 0.095171065 | 0.302629329 |
| **DNAJA4** | 0.378033075 | 0.886294841 | 0.564749663 | 0.467758485 | 0.121531745 | 0.150752236 |
| **SPP1** | 0.349558921 | -0.093392606 | -0.248980381 | 0.54270041 | 0.395140269 | 1.692273826 |
| **MARCHF3** | 0.38422092 | 0.125211439 | 0.086728183 | 0.386087554 | 0.045845275 | 0.579307814 |
| **CASQ1** | 1.288746378 | 1.135706539 | -0.046454323 | 0.157237922 | -0.527111402 | 0.125906028 |
| **ODF2** | 0.651289686 | 0.023944301 | 0.374517645 | 0.343318321 | 0.134278009 | 0.295319489 |
| **FCGBP** | 0.438433511 | -0.309801042 | NA | 0.195296203 | 0.900287722 | 0.538694691 |
| **EGLN3** | 0.74278394 | 1.120847355 | -0.167523324 | 0.159125662 | -0.464453082 | 0.245513257 |
| **TGFBI** | 0.546478841 | 0.457803125 | -0.097927633 | 0.562117959 | 0.892872631 | 0.389591235 |
| **C1QB** | 0.630226382 | NA | -0.064146243 | 0.426778558 | 1.463149715 | -0.452962005 |
| **CCND1** | 0.174657557 | 1.146196248 | 0.021776354 | 0.516685175 | -0.054146919 | 0.018956803 |
| **ODC1** | 0.365612507 | 1.059261039 | -0.179051418 | 0.347688781 | NA | 0.057191541 |
| **LRRC2** | -0.069161138 | 0.447587741 | 0.549733196 | 0.493894464 | -0.461994392 | 0.870977251 |
| **ASB11** | 0.622966504 | 0.314163945 | 0.975633956 | 0.028509264 | NA | NA |
| **CPLX1** | 0.619709535 | 0.206505045 | 0.067040238 | 0.729562731 | -0.076942632 | NA |
| **S100A8** | 0.178810654 | -1.137182876 | 0.766665214 | 0.013551084 | 2.08245821 | 0.262264726 |
| **SERPINE2** | 0.360073156 | 1.006402744 | NA | 0.304440256 | 0.154298788 | 0.172222351 |
| **FHL1** | 0.616858045 | 1.518979392 | 0.260613073 | -0.221167732 | 0.549691945 | -0.204811566 |
| **RGS18** | 0.236869282 | 0.388334738 | 1.000140647 | -0.002815482 | 1.187228749 | NA |
| **NPC2** | 0.627479006 | 0.33858635 | 0.204846533 | 0.300437486 | 1.052804061 | -0.01557775 |
| **GPR34** | -0.163661954 | 0.079513565 | 0.7413379 | 0.10988151 | 1.274117714 | NA |
| **TMEM45A** | 1.025229068 | 0.232374751 | 0.198733146 | 0.375704597 | 0.432359952 | 0.486291325 |
| **LOXL1** | 0.401653483 | 0.942610032 | NA | 0.724856324 | 0.857537014 | 0.348454718 |
| **COL5A2** | 0.60364224 | 1.135981748 | 0.172103156 | 0.430007402 | 0.184803109 | 0.274975012 |
| **NPR3** | 0.447784333 | 2.603388872 | NA | 0.204239659 | 0.598790761 | -0.517892499 |
| **HCG11** | -0.154492835 | 1.5495638 | 0.7345109 | 0.185464139 | NA | NA |
| **SLC6A6** | 0.495301228 | 0.323779623 | 0.515320979 | 0.427230651 | 0.296154195 | 0.193970084 |
| **TGFB1I1** | 0.807461875 | -0.259681912 | 0.115003555 | 0.292384055 | 0.496877783 | 0.446269974 |
| **EVI2B** | 0.280757226 | -0.042089044 | 0.72675429 | 0.118615701 | 1.250501358 | -0.088475225 |
| **MIF** | 0.67092336 | 0.276885144 | -0.063299695 | 0.159521562 | 1.312216368 | 0.446892688 |
| **SNAI2** | 0.358237652 | 1.172878671 | 0.688268734 | 0.17395452 | 0.843248999 | -0.19821042 |
| **RPS6KA2** | 0.389569078 | -0.198123448 | 0.05042457 | 0.289537832 | 0.232857987 | 0.330142214 |
| **RABAC1** | 0.585657128 | 0.303850968 | 0.146828084 | 0.099804865 | 1.2025937 | 0.091424329 |
| **ADCY6** | 0.44065832 | 0.576427949 | 0.1782086 | 0.328052383 | 0.022959226 | 0.454136952 |
| **FIBP** | 0.304768072 | 0.60440083 | -0.02624985 | 0.350678249 | 0.615387181 | 0.168265781 |
| **THY1** | 0.494731812 | 0.716707402 | NA | 0.451549889 | 0.248856837 | 0.510867239 |
| **TP53INP2** | 0.433326302 | 0.675965626 | -0.224617267 | 0.276060773 | NA | NA |
| **OSTF1** | 0.310102185 | 0.829420774 | 0.239034968 | 0.054061148 | 1.115740446 | 0.16055699 |
| **PCDH20** | 1.674947068 | -0.284039939 | -0.165278521 | 0.136989453 | -1.457657604 | NA |
| **LOC105373170** | -0.876167375 | 2.52323071 | -0.11058434 | -0.619536845 | -0.751771981 | NA |
| **ABTB2** | 0.449022061 | -0.194091289 | -0.098145742 | 0.274620565 | -0.171330073 | 0.297144214 |
| **DOK5** | 0.322320829 | 0.753902038 | NA | 0.270841124 | -0.187951804 | 0.124362084 |
| **BNIP3** | 0.069843046 | 0.550678806 | 0.356851848 | 0.401744563 | 0.130023184 | 0.444241292 |
| **C1QA** | 0.55045835 | 0.374394856 | 0.025631819 | 0.227132025 | 1.493703976 | -0.451418112 |
| **CXCL12** | -0.345120699 | NA | 0.517603256 | 0.370240631 | 0.808223452 | -0.204582183 |
| **OXCT1** | 0.212716993 | 0.172084445 | 0.247163009 | 0.515335516 | 0.142766546 | 0.410525074 |
| **CDH2** | 0.104303687 | 0.966450808 | 0.525638919 | 0.266684507 | -0.081314809 | 0.1029782 |
| **GPX1** | 0.416349374 | 0.070249651 | 0.068537595 | -0.02302679 | 1.082039569 | 0.254937792 |
| **CHST11** | 0.344260487 | -0.552057019 | 0.146609533 | 0.307403492 | 0.158993667 | 0.254052725 |
| **GRIP2** | NA | -0.183210303 | 0.160587472 | 0.427172093 | -0.056902926 | 0.407816349 |
| **DNER** | 1.45346491 | 0.701421829 | -1.160800177 | -0.84198743 | -2.448248954 | NA |
| **EGR1** | 0.355497394 | 2.293488852 | -0.085989618 | -0.641263283 | 0.575185902 | -0.16954708 |
| **FAM216B** | -0.599417993 | -1.020949594 | 1.39722306 | -0.213162296 | 0.433363733 | NA |
| **S100A9** | -0.054070273 | -1.981802268 | 0.535652279 | 0.041907807 | 2.053068613 | 0.182593123 |
| **RGS2** | 0.640126462 | 0.702724966 | -0.055601576 | -0.056712 | 1.051110306 | -0.280405466 |
| **COL15A1** | 0.109992479 | 0.932388866 | 0.769026043 | 0.16371039 | 0.662114604 | -0.146826134 |
| **UBE2M** | 0.107047371 | 0.343388882 | 0.817803604 | 0.116974104 | 1.047445624 | 0.097547474 |
| **LAPTM5** | 0.32356465 | -0.030491245 | 0.141217105 | 0.423143712 | 1.34412695 | -0.180134758 |
| **SERPINH1** | 0.670245253 | 0.923952139 | 0.340022964 | 0.230751343 | 0.209760569 | 0.22301964 |
| **ESM1** | 0.19383261 | 0.953560715 | 0.223235098 | 0.165415937 | -0.212024557 | 0.381761678 |
| **ENO2** | 0.901899127 | 0.288714341 | -0.202213893 | 0.422582824 | 0.034793905 | 0.690363369 |
| **YKT6** | 0.288233782 | 0.574731087 | 0.130868856 | 0.335199641 | 0.575430769 | 0.083366621 |
| **NCF2** | -0.165719044 | -0.238473753 | 0.681212474 | 0.146210888 | 1.554763921 | 0.081758054 |
| **HLA-DRA** | -0.387679891 | 0.703932142 | 0.678743341 | 0.427358402 | 1.422623827 | -0.398028506 |
| **PDGFD** | 0.306771399 | 0.347222704 | 0.677781412 | 0.253210143 | 0.922413338 | 0.190055724 |
| **FRZB** | 0.209992101 | 0.580958482 | 0.469542584 | 0.271057003 | 0.40834943 | 0.732140744 |
| **PAX8-AS1** | 0.573067763 | 0.914922976 | -0.165999284 | 0.04133283 | -0.307133526 | NA |
| **CLIC4** | 0.373451947 | 0.3566679 | 0.479131877 | 0.01733669 | 0.764864701 | -0.388591101 |
| **CEBPD** | 0.507419055 | 0.174846295 | -0.100660483 | -0.055894156 | 1.088115406 | -0.226573443 |
| **DNAJB4** | 0.022897878 | 0.912348388 | 0.916605504 | 0.05595777 | -0.232839828 | 0.046528692 |
| **C16orf54** | -0.03050197 | -0.223252727 | 0.826379094 | 0.047150772 | 1.036759146 | NA |
| **HBA2** | 0.827590981 | 2.232562318 | -0.134411677 | 0.176070868 | 0.956297775 | 0.146303318 |
| **B3GALT2** | -0.642925633 | -0.17205855 | 1.287521946 | -0.271627473 | -0.159458082 | -0.374361707 |
| **FCER1G** | 0.059074455 | -1.029939469 | 0.340269618 | 0.268927582 | 1.878134893 | -0.108033408 |
| **ST3GAL1** | 0.502978914 | 0.232429418 | -0.194183743 | 0.444929751 | -0.103706957 | 0.10337147 |
| **ZBTB6** | -0.197794924 | 0.903442882 | 0.752218026 | -0.021946881 | -0.218683328 | -0.024404218 |
| **CORO1A** | 0.400554192 | 0.476563183 | 0.256235585 | 0.421551662 | 1.024222874 | -0.032844929 |
| **GGT5** | 0.394694991 | 0.097287903 | 0.185741702 | 0.221376834 | 0.75580908 | 0.272792221 |
| **LTBP1** | 0.751954891 | 0.517933906 | -0.107835933 | 0.403459289 | 0.101708486 | -0.202137014 |
| **PPBP** | 0.495537619 | NA | 0.851706135 | -0.100091377 | 0.426484067 | 0.129152061 |
| **TYROBP** | 0.489631218 | -0.142581386 | 0.380318439 | 0.374613956 | 1.861394973 | -0.219725987 |
| **HEY1** | 0.125229789 | 0.981970454 | 0.265790896 | 0.224442074 | 0.382813163 | 0.355721995 |
| **WASHC2C** | -0.537872629 | -0.425658642 | -0.41247325 | -0.410201335 | -0.373487797 | -0.710679562 |
| **CYP8B1** | -0.25040769 | -0.108139413 | -0.137323509 | -0.02158831 | -0.131828372 | NA |
| **RNASEH2C** | 0.080133826 | -0.250134521 | -0.056340679 | -0.010761121 | 0.299181509 | NA |
| **TBC1D2** | 0.050681633 | -0.420231508 | -0.25006848 | 0.153997372 | 0.378126802 | 0.072166034 |
| **PRPH2** | -0.046924086 | -0.464676479 | -0.399986493 | -0.250539808 | -0.068999145 | 0.048315757 |
| **ADAM21** | -0.023398445 | -0.007431311 | -0.06993761 | 0.009085649 | -0.250117682 | 0.084432267 |
| **ATP6V0E2** | -0.303947178 | -0.145100397 | NA | -0.127637935 | 0.041431918 | -0.250324067 |
| **USP54** | -0.353484783 | -0.351913281 | -0.355582291 | -0.399869517 | -0.086371238 | NA |
| **TNFSF13** | 0.177988194 | 0.038743899 | -0.254259835 | -0.035825706 | 0.565157452 | -0.257566704 |
| **PCP4** | -0.263050513 | -0.135388335 | -0.463026308 | -0.283572341 | -0.599842025 | -0.061409465 |
| **KDM7A-DT** | -0.25810256 | -0.067748235 | NA | 0.131046353 | -0.256052622 | NA |
| **BCL6** | -0.016556446 | NA | 0.030248748 | -0.285494508 | 0.3020739 | -0.258252505 |
| **MOCS2** | -0.251660333 | 0.128509388 | 0.210564767 | -0.087618622 | -0.216466692 | -0.111453662 |
| **KCNIP4** | -0.006045281 | -0.25037501 | -0.055422369 | 0.007534758 | 0.10992001 | NA |
| **KRT38** | -0.150085822 | -0.23435315 | -0.250241727 | -0.073346405 | -0.15064984 | 0.186150981 |
| **NINL** | -0.089600604 | -0.468128761 | -0.415634268 | -0.250845221 | -0.431030394 | -0.077471381 |
| **ZNF738** | -0.21698896 | -0.247691049 | 0.213128659 | -0.153713033 | -0.250286502 | NA |
| **GLG1** | 0.212979351 | 0.513741324 | -0.159956545 | 0.195511472 | -0.019976078 | -0.250374772 |
| **BMP7** | -0.067784024 | 0.026165293 | -0.288342277 | -0.272597586 | -0.37793644 | -0.302314229 |
| **CRTAC1** | -0.482600354 | -0.345364164 | -0.434054572 | -0.341247552 | -0.31818136 | -0.083152862 |
| **CLCN3** | -0.252750258 | 0.162266916 | 0.383007741 | 0.073079522 | -0.137216032 | -0.141153195 |
| **ZFP92** | -0.146740712 | -0.252179071 | -0.093502135 | 0.037604347 | -0.034268664 | NA |
| **TRIM71** | 0.033273618 | -0.052536528 | -0.250334832 | -0.023626775 | NA | NA |
| **RPL23AP32** | -0.042563267 | -0.402067409 | -0.269572791 | -0.144538904 | -0.040223727 | -0.250590815 |
| **MLF1** | -0.286876117 | NA | 0.479170717 | -0.251559962 | -0.358507646 | -0.233923853 |
| **ST8SIA2** | 0.19936979 | 0.699676193 | -0.04715762 | 0.050125364 | -0.250607576 | 0.160676696 |
| **SLC6A16** | -0.132611309 | -0.91642189 | -0.263137857 | -0.289429513 | -0.113306077 | 0.098408771 |
| **KRT10-AS1** | -0.253540514 | -0.122744121 | -0.067861722 | 0.061760362 | 0.035743581 | NA |
| **ARAP1** | 0.121781841 | -0.253034982 | -0.026629881 | -0.042465948 | 0.103380521 | -0.030643095 |
| **MYRIP** | -0.011657086 | -0.171494591 | -0.250368862 | -0.149668331 | 0.04529564 | -0.060558749 |
| **PLAG1** | -0.356230239 | -0.335671989 | 0.258974722 | -0.252258597 | -0.805751422 | -0.073265536 |
| **LOC101927709** | 0.060615677 | -0.11347119 | NA | -0.02348351 | -0.250758087 | NA |
| **SUZ12** | -0.047794898 | -0.07861525 | -0.0950951 | -0.003685163 | -0.162801624 | -0.250648374 |
| **SUSD4** | -0.651819655 | NA | -0.541190185 | -0.638095565 | -0.730689867 | -0.549999837 |

## Supplementary Table 2. Genes related to atrial fibrillation browsed in Phenolyzer.

| **Rank** | **Gene** | **ID** | **Score** | **HaploinsufficiencyScore** | **GeneIntoleranceScore** |
| --- | --- | --- | --- | --- | --- |
| 1 | *KCNJ4* | 3761 | 0.155 | 0.171 | 0.896968625 |
| 2 | *ADCY6* | 112 | 0.1098 | 0.569 | 0.94096485 |
| 3 | *ATP1B4* | 23439 | 0.08536 | 0.149 | 0.342415664 |
| 4 | *KCNIP4* | 80333 | 0.08331 | 0.321 | 0.699339467 |
| 5 | *TNNT1* | 7138 | 0.06949 | 0.612 | 0.5351498 |
| 6 | *YWHAQ* | 10971 | 0.06863 | 0.985 | 0.522116065 |
| 7 | *RPS6KA2* | 6196 | 0.0667 | 0.521 | 0.918553904 |
| 8 | *FGF1* | 2246 | 0.06333 | 0.923 | 0.45051899 |
| 9 | *HSP90AB1* | 3326 | 0.05765 | 0.149 | 0.947629158 |
| 10 | *EGR1* | 1958 | 0.05341 | 0.989 | 0.889419675 |
| 11 | *COL4A2* | 1284 | 0.05223 | 0.239 | 0.834689785 |
| 12 | *COL4A1* | 1282 | 0.05217 | 0.828 | 0.993807502 |
| 13 | *CCND1* | 595 | 0.05138 | 0.999 | 0.68542109 |
| 14 | *CEBPD* | 1052 | 0.04994 | 0 | 0 |
| 15 | *CDH2* | 1000 | 0.0479 | 0.934 | 0.924805379 |
| 16 | *ST8SIA2* | 8128 | 0.04617 | 0.33 | 0.744397264 |
| 17 | *SUZ12* | 23512 | 0.04269 | 0.843 | 0.58091531 |
| 18 | *RGS2* | 5997 | 0.04163 | 0.173 | 0.583569238 |
| 19 | *TYROBP* | 7305 | 0.03898 | 0.122 | 0.310155697 |
| 20 | *IGFBP3* | 3486 | 0.03796 | 0.274 | 0 |
| 21 | *CXCR4* | 7852 | 0.03604 | 0.888 | 0.673802784 |
| 22 | *SLC25A5* | 292 | 0.0357 | 0.306 | 0.489620193 |
| 23 | *HSPA2* | 3306 | 0.03507 | 0.653 | 0.366418967 |
| 24 | *ATP6V0E2* | 155066 | 0.03444 | 0 | 0 |
| 25 | *SPP1* | 6696 | 0.03353 | 0.492 | 0.496579382 |
| 26 | *COL1A2* | 1278 | 0.03195 | 0.999 | 0.936364709 |
| 27 | *THY1* | 7070 | 0.03111 | 0.688 | 0.453703704 |
| 28 | *BCL6* | 604 | 0.03101 | 0.285 | 0.764272234 |
| 29 | *DOK5* | 55816 | 0.03009 | 0.381 | 0.815581505 |
| 30 | *KRT38* | 8687 | 0.02978 | 0.072 | 0.691790517 |
| 31 | *DPYSL4* | 10570 | 0.02953 | 0.2 | 0.964319415 |
| 32 | *ST3GAL1* | 6482 | 0.02869 | 0.106 | 0.285031847 |
| 33 | *RPL3L* | 6123 | 0.02853 | 0.862 | 0.131457891 |
| 34 | *GPX1* | 2876 | 0.0282 | 0.282 | 0.310155697 |
| 35 | *ACTA1* | 58 | 0.028 | 0.876 | 0.673802784 |
| 36 | *THBS2* | 7058 | 0.02334 | 0.746 | 0.769521114 |
| 37 | *PPBP* | 5473 | 0.02301 | 0.268 | 0.571243218 |
| 38 | *NPPB* | 4879 | 0.023 | 0.054 | 0.19420854 |
| 39 | *ENO2* | 2026 | 0.02297 | 0.815 | 0.73767398 |
| 40 | *RCAN1* | 1827 | 0.02249 | 0.141 | 0.502418023 |
| 41 | *DGKI* | 9162 | 0.02243 | 0.083 | 0.922269403 |
| 42 | *WIPI1* | 55062 | 0.02194 | 0.198 | 0.366418967 |
| 44 | *BGN* | 633 | 0.02139 | 0.973 | 0.58091531 |
| 45 | *YKT6* | 10652 | 0.02118 | 0.111 | 0.68542109 |
| 46 | *CYP8B1* | 1582 | 0.02095 | 0.123 | 0.730714791 |
| 47 | *TIMP1* | 7076 | 0.02083 | 0.447 | 0.417374381 |
| 48 | *MIF* | 4282 | 0.02042 | 0.166 | 0 |
| 49 | *FHL2* | 2274 | 0.02024 | 0.422 | 0.277718802 |
| 50 | *HEY1* | 23462 | 0.02018 | 0.427 | 0.516513329 |
| 51 | *COL1A1* | 1277 | 0.01962 | 0.992 | 0.977412126 |
| 52 | *IGFBP2* | 3485 | 0.01885 | 0.831 | 0 |
| 53 | *ODC1* | 4953 | 0.01879 | 0.228 | 0.756664308 |
| 54 | *MYRIP* | 25924 | 0.01856 | 0.32 | 0.857572541 |
| 55 | *ALYREF* | 10189 | 0.01832 | 0 | 0 |
| 56 | *COL3A1* | 1281 | 0.01828 | 0.997 | 0.636588818 |
| 57 | *NCF2* | 4688 | 0.01798 | 0.102 | 0.059860816 |
| 58 | *GGT5* | 2687 | 0.01784 | 0.045 | 0.717975938 |
| 59 | *MOCS2* | 4338 | 0.01766 | 0.06 | 0.192675159 |
| 60 | *HLA-DRA* | 3122 | 0.0176 | 0.123 | 0.27990092 |
| 61 | *HBA2* | 3040 | 0.01732 | 0.073 | 0 |
| 62 | *TNFSF13* | 8741 | 0.01715 | 0 | 0.344420854 |
| 63 | *TGFBI* | 7045 | 0.01698 | 0.477 | 0.839761736 |
| 64 | *CPLX1* | 10815 | 0.01689 | 0 | 0.587520642 |
| 65 | *RELN* | 5649 | 0.01684 | 0.202 | 0.985373909 |
| 66 | *CXCL12* | 6387 | 0.01669 | 0.543 | 0.45051899 |
| 67 | *GRIP2* | 80852 | 0.01633 | 0.312 | 0 |
| 68 | *NPC2* | 10577 | 0.01627 | 0.165 | 0.289219156 |
| 69 | *CLIC4* | 25932 | 0.01599 | 0.399 | 0.631398915 |
| 70 | *RGS4* | 5999 | 0.01597 | 0.132 | 0.518813399 |
| 71 | *RGS18* | 64407 | 0.01597 | 0.213 | 0.268872376 |
| 72 | *CHST11* | 50515 | 0.0156 | 0.247 | 0.723106865 |
| 73 | *NINL* | 22981 | 0.01531 | 0 | 0.050601557 |
| 74 | *EGLN3* | 112399 | 0.01524 | 0.752 | 0.518813399 |
| 75 | *COL21A1* | 81578 | 0.01451 | 0.445 | 0.141483841 |
| 76 | *CXCR2* | 3579 | 0.01424 | 0 | 0.172564284 |
| 77 | *UBE2M* | 9040 | 0.01407 | 0.275 | 0.372611465 |
| 78 | *ZNF738* | 148203 | 0.01396 | 0.078 | 0 |
| 79 | *CES2* | 8824 | 0.01343 | 0.062 | 0.891542817 |
| 80 | *OXCT1* | 5019 | 0.01331 | 0.084 | 0.611818825 |
| 81 | *SNAI2* | 6591 | 0.01317 | 0.296 | 0.537980656 |
| 82 | *DHRS9* | 10170 | 0.01313 | 0.146 | 0.154458599 |
| 83 | *FCER1G* | 2207 | 0.0127 | 0.126 | 0.262149092 |
| 84 | *P2RX5* | 5026 | 0.0125 | 0.06 | 0.165251239 |
| 85 | *SLC6A6* | 6533 | 0.01237 | 0.264 | 0.667964143 |
| 86 | *CLCN3* | 1182 | 0.01204 | 0.412 | 0.814107101 |
| 87 | *COL5A1* | 1289 | 0.01142 | 0.627 | 0.994751121 |
| 88 | *COL5A2* | 1290 | 0.01142 | 0.408 | 0.894078792 |
| 89 | *CASQ1* | 844 | 0.0113 | 0.445 | 0.172564284 |
| 90 | *COLEC11* | 78989 | 0.01123 | 0.13 | 0.547416844 |
| 91 | *CRLF1* | 9244 | 0.01112 | 0.346 | 0.836400094 |
| 92 | *OSTF1* | 26578 | 0.01042 | 0.127 | 0.66029724 |
| 93 | *ODF2* | 4957 | 0.01029 | 0.425 | 0.846367068 |
| 94 | *PDGFD* | 80310 | 0.01026 | 0.183 | 0.794644963 |
| 95 | *TRIM71* | 131405 | 0.0102 | 0.307 | 0.935656995 |
| 96 | *FRZB* | 2487 | 0.01005 | 0.207 | 0.222045294 |
| 97 | *COL15A1* | 1306 | 0.00952 | 0.183 | 0.028957301 |
| 98 | *C1QB* | 713 | 0.009211 | 0.147 | 0.499882048 |
| 99 | *C1QA* | 712 | 0.009211 | 0.164 | 0.567055909 |
| 100 | *S100A8* | 6279 | 0.008911 | 0.23 | 0.437485256 |
| 101 | *S100A9* | 6280 | 0.008911 | 0.399 | 0.387237556 |
| 102 | *PHLDA1* | 22822 | 0.00873 | 0.284 | 0 |
| 103 | *B3GALT2* | 8707 | 0.008425 | 0.234 | 0.656817646 |
| 104 | *S100A12* | 6283 | 0.008351 | 0.014 | 0.489620193 |
| 105 | *MNDA* | 4332 | 0.007567 | 0.079 | 0.326963907 |
| 106 | *QPCT* | 25797 | 0.007567 | 0.288 | 0.274003303 |
| 107 | *ARAP1* | 116985 | 0.007496 | 0 | 0.960662892 |
| 108 | *ASB11* | 140456 | 0.007204 | 0.145 | 0.353916018 |
| 109 | *RNF216* | 54476 | 0.007127 | 0.102 | 0.941082803 |
| 110 | *PXDN* | 7837 | 0.006646 | 0.346 | 0.965204058 |
| 111 | *LTBP1* | 4052 | 0.00652 | 0.459 | 0.755307856 |
| 112 | *BMP7* | 655 | 0.006367 | 0.994 | 0.785857514 |
| 113 | *COMP* | 1311 | 0.005872 | 0.623 | 0.773531493 |
| 114 | *PRICKLE1* | 144165 | 0.005809 | 0.737 | 0.665251239 |
| 115 | *TBC1D2* | 55357 | 0.005772 | 0.078 | 0.314166077 |
| 116 | *DNER* | 92737 | 0.005707 | 0.23 | 0.918966737 |
| 117 | *NPR3* | 4883 | 0.005459 | 0.644 | 0.462727058 |
| 118 | *MXRA5* | 25878 | 0.005396 | 0.211 | 0.043170559 |
| 119 | *ALPK3* | 57538 | 0.005396 | 0.133 | 0.314519934 |
| 120 | *GLG1* | 2734 | 0.005248 | 0.151 | 0.973460722 |
| 121 | *SERPINE2* | 5270 | 0.004885 | 0.223 | 0.734666195 |
| 122 | *CHGB* | 1114 | 0.003877 | 0.186 | 0.024946921 |
| 123 | *BNIP3* | 664 | 0.003662 | 0.141 | 0.46809389 |
| 124 | *ETV5* | 2119 | 0.002967 | 0.597 | 0.708362821 |
| 125 | *LTBP2* | 4053 | 0.002834 | 0.309 | 0.967091295 |
| 126 | *LOXL1* | 4016 | 0.002821 | 0.903 | 0 |
| 127 | *SLC6A16* | 28968 | 0.002395 | 0.057 | 0.817881576 |
| 128 | *TGFB1I1* | 7041 | 0.001986 | 0.756 | 0.767456947 |
| 129 | *SERPINH1* | 871 | 0.001757 | 0.778 | 0.820889361 |
| 130 | *ASPN* | 54829 | 0.001716 | 0.718 | 0 |
| 131 | *MLF1* | 4291 | 0.001012 | 0.193 | 0.192675159 |
| 132 | *PLAG1* | 5324 | 0.001009 | 0.767 | 0.628862939 |
| 133 | *EVI2B* | 2124 | 0.0004163 | 0.084 | 0.229358339 |
| 134 | *NRAP* | 4892 | 0.0002797 | 0.05 | 0.007254069 |
| 135 | *FHL1* | 2273 | 0.0002062 | 0.426 | 0.485963671 |
| 136 | *ZFP92* | 139735 | 0.0001862 | 0.069 | 0 |
| 137 | *CORO1A* | 11151 | 9.24E-05 | 0.785 | 0.785857514 |
| 138 | *GPR34* | 2857 | 5.60E-05 | 0.26 | 0.433592829 |
| 139 | *PCDH20* | 64881 | 3.38E-06 | 0.268 | 0.907289455 |
| 140 | *DNAJA4* | 55466 | 3.38E-06 | 0.137 | 0.160238264 |
| 141 | *DNAJB4* | 11080 | 3.38E-06 | 0.545 | 0.68542109 |
| 142 | *USP54* | 159195 | 2.95E-11 | 0.21 | 0.086223166 |
| 143 | *PAX8-AS1* | 654433 | 2.61E-11 | 0 | 0 |
| 144 | *HCG11* | 493812 | 2.61E-11 | 0 | 0 |
| 145 | *UNC5B-AS1* | 728978 | 2.61E-11 | 0 | 0 |
| 146 | *PLEKHO1* | 51177 | 2.27E-11 | 0.085 | 0.765746638 |
| 147 | *LAPTM5* | 7805 | 9.50E-12 | 0.132 | 0.28591649 |
| 148 | *TP53INP2* | 58476 | 2.86E-14 | 0.213 | 0 |
| 149 | *ABTB2* | 25841 | 5.68E-21 | 0.353 | 0.918259023 |
| 150 | *MPV17* | 4358 | 1.40E-62 | 0.11 | 0.66029724 |

**The followings are genes out of Phenolyzer results:** *"COLQ, LDAF1, LBH, MTCL1, MAP3K7CL, TRDN-AS1, DIRAS3, DPT, ANGPTL2, LOC101928304, SPRY4, NES, HOOK2, CLC, PCSK1N, POPDC3, LOC101927069, HK2, ATP13A3, MARCHF3, FCGBP, LRRC2, TMEM45A, RABAC1, FIBP, LOC105373170, FAM216B, ESM1, C16orf54, ZBTB6, WASHC2C, RNASEH2C, PRPH2, ADAM21, PCP4, KDM7A-DT, CRTAC1, RPL23AP32, KRT10-AS1, LOC101927709, SUSD4".*

## Supplementary Table 3. Top 50 results of CMap analysis.

| **Score** | **ID** | **Name** | **Target** | **MOA** |
| --- | --- | --- | --- | --- |
| **-95.96** | BRD-K12244279 | MEK1-2-inhibitor | *MAP2K1, MAP2K2* | MEK inhibitor |
| **-93.66** | BRD-U73238814 | QL-XI-92 | *DDR1* | DDR1 inhibitor |
| **-91.34** | BRD-K56343971 | vemurafenib | *BRAF, CYP2C19, CYP3A4, CYP3A5, RAF1* | RAF inhibitor |
| **-90.04** | BRD-K36740062 | GSK-1070916 | *AURKB, AURKC, AURKA, CYP2D6, CYP3A4* | Aurora kinase inhibitor |
| **-88.35** | BRD-K86930074 | cediranib | *KDR, FLT1, FLT4, KIT, PDGFRB, CSF1R, FLT3, PDGFRA* | KIT inhibitor, VEGFR inhibitor |
| **-87.88** | BRD-K20285085 | fostamatinib | *SYK, FLT3, RET* | SYK inhibitor |
| **-86.98** | BRD-K66766661 | 17-beta-estradiol | *ESR1* | Estrogen receptor agonist |
| **-85.81** | BRD-K57080016 | selumetinib | *MAP2K1, MAP2K2* | MEK inhibitor |
| **-85.69** | BRD-K95785537 | PP-2 | *SRC, LCK, ABL1, LYN, RIPK2* | SRC inhibitor |
| **-84.06** | BRD-K18787491 | U-0126 | *AKT1, CHEK1, GSK3B, JAK2, LCK, MAP2K1, MAP2K2, MAP2K7, MAPK1, MAPK11, MAPK12, MAPK14, MAPK8, PRKCA, RAF1, ROCK1, RPS6KB1, SGK1* | MEK inhibitor |
| **-82.88** | BRD-K05104363 | PD-184352 | *MAP2K1, MAP2K2, MAP3K1, MAP3K2* | MEK inhibitor |
| **-77.74** | BRD-A25687296 | emetine | *RPS2* | Protein synthesis inhibitor |
| **-77.36** | BRD-A26002865 | verrucarin-a |  | Protein synthesis inhibitor |
| **-77.19** | BRD-A17411484 | carprofen | *PTGS2, PTGS1* | Cyclooxygenase inhibitor |
| **-76.91** | BRD-K84085265 | CG-930 | *MAPK8* | JNK inhibitor |
| **-75.97** | BRD-K80348542 | cephaeline | *RPS2* | Protein synthesis inhibitor |
| **-74.22** | BRD-K99964838 | bosutinib | *ABL1, SRC, BCR, CAMK2G, LYN, CAMK1D, CDK2, FRK, FYN, HCK, MAP2K1, MAP2K2, MAP3K2, MAP4K5, STK10, STK24, STK4, TNK2, TXK* | ABL inhibitor, BCR-ABL kinase inhibitor, SRC inhibitor |
| **-73.86** | BRD-K76674262 | homoharringtonine | *RPL3* | Protein synthesis inhibitor |
| **-73.41** | BRD-A17428743 | BW-723C86 | *HTR2B, HTR2A, HTR2C* | Serotonin receptor agonist |
| **-73.38** | BRD-A41112154 | oleanolic-acid |  | G protein-coupled receptor agonist |
| **-72.26** | BRD-K32107296 | temozolomide | *TOP2A* | DNA alkylating agent |
| **-72.11** | BRD-K47659338 | EMD-386088 | *HTR6* | Serotonin receptor agonist |
| **-71.85** | BRD-K50168500 | canertinib | *EGFR, ERBB2, ERBB4, AKT1* | EGFR inhibitor |
| **-71.52** | BRD-K19525698 | ozagrel | *TBXAS1* | Thromboxane synthase inhibitor |
| **-71.49** | BRD-K41895714 | AS-605240 | *MAOB, PIK3CA, PIK3CB, PIK3CD, PIK3CG* | PI3K inhibitor |
| **-69.08** | BRD-K74236984 | UNC-0321 | *EHMT2* | Histone lysine methyltransferase inhibitor |
| **-69.02** | BRD-K77793136 | hydroxyfasudil | *ROCK1, PKIA, PRKACA, ROCK2* | Rho associated kinase inhibitor |
| **-67.46** | BRD-K67566344 | KU-0063794 | *MTOR* | MTOR inhibitor |
| **-67.16** | BRD-A29644307 | nomifensine | *SLC6A2, SLC6A3, DRD2, MAOA, MAOB, MPO* | Dopamine uptake inhibitor, Noradrenaline uptake inhibitor |
| **-66.65** | BRD-K05804044 | AZ-628 | *BRAF, RAF1* | RAF inhibitor |
| **-66.3** | BRD-K73293050 | WZ-3146 | *EGFR* | EGFR inhibitor |
| **-66.19** | BRD-K66175015 | afatinib | *EGFR, ERBB2, ERBB4* | EGFR inhibitor |
| **-65.47** | BRD-A75479906 | rimantadine |  | Antiviral, RNA synthesis inhibitor |
| **-65.46** | BRD-K37720887 | SB-525334 | *TGFBR1* | TGF beta receptor inhibitor |
| **-65.22** | BRD-K79404599 | enzastaurin | *PRKCB, AKT1, GSK3B, PRKCA, PRKCD, PRKCG* | PKC inhibitor |
| **-64.47** | BRD-A24122750 | saclofen | *GABBR1, GABBR2, KCTD12, KCTD16, KCTD8* | GABA receptor antagonist |
| **-64.35** | BRD-K17294426 | clebopride | *DRD2, ACHE* | Dopamine receptor antagonist |
| **-63.5** | BRD-K52751261 | TAK-715 | *MAPK14, TNF* | p38 MAPK inhibitor |
| **-62.95** | BRD-K89014967 | AS-703026 | *MAP2K1, MAP2K2* | MEK inhibitor |
| **-62.22** | BRD-K70914287 | BIBX-1382 | *EGFR, ERBB2* | EGFR inhibitor, Tyrosine kinase inhibitor |
| **-61.94** | BRD-K15588452 | R-96544 | *HTR2A* | Serotonin receptor antagonist |
| **-61.42** | BRD-K02965346 | SU-11274 | *MET* | Hepatocyte growth factor receptor inhibitor, Tyrosine kinase inhibitor |
| **-59.77** | BRD-K49865102 | PD-0325901 | *MAP2K1, MAP2K2* | MEK inhibitor, MAP kinase inhibitor, Protein kinase inhibitor |
| **-59.23** | BRD-A24228527 | ofloxacin | *TOP2A* | Bacterial DNA gyrase inhibitor |
| **-57.95** | BRD-K15791587 | L-733060 | *TACR1* | Tachykinin antagonist |
| **-57.43** | BRD-K60690191 | MPEP | *GRM5, GRM1, GRM4* | Glutamate receptor antagonist |
| **-57.13** | BRD-K06750613 | GSK-1059615 | *PIK3CA, PIK3CG* | PI3K inhibitor |
| **-56.83** | BRD-K88677950 | PD-198306 | *MAP2K1, MAP2K2, MAPK1, MAPK3* | MAP kinase inhibitor, MEK inhibitor |
| **-56.52** | BRD-A94793051 | gestrinone | *PGR, AR, ESR1* | Progesterone receptor antagonist |
| **-54.66** | BRD-K32836707 | CAY-10577 |  | Casein kinase inhibitor |

## Supplementary Table 4. Main parameters of diagnostic model.

| The main Model Parameters are shown as follows | | | | | | | | |
| --- | --- | --- | --- | --- | --- | --- | --- | --- |
| learning_rate | n_estimators | max_depth | min_child_weight | gamma | subsample | colsample_bytree | objective | scale_pos_weight |
| 0.08 | 400 | 4 | 0 | 0.0 | 0.6 | 0.8 | binary:logistic | 1 |

## Supplementary Table 5. MCODE.

| **Cluster** | **Score (Density*#Nodes)** | **Nodes** | **Edges** | **Node IDs** |
| --- | --- | --- | --- | --- |
| 1 | 5.6 | 6 | 28 | *ADAMTS2, SERPINH1, COL5A2, COL21A1, COL4A2, COL5A1* |
| 2 | 3 | 3 | 6 | *NPPB, NPPA, RCAN1* |
| 3 | 3 | 3 | 6 | *HSPA4, DNAJB4, DNAJA4* |
| 4 | 3 | 3 | 6 | *CTNNA1, CDH2, CTNND2* |

**Network Scoring: Include Loops: false Degree Cutoff: 2; Cluster Finding: Node Score Cutoff: 0.2; Haircut: true; Fluff: false; K-Core: 2; Max. Depth from Seed: 100**

## Supplementary Table 6. The distinctive cytokine-related different expressed genes among different clusters.

| Names | total | elements |
| --- | --- | --- |
| Cluster2 vs Cluster 1 | **10** | **CXCL3, INSL4, CGA, CHGA, EDN2, GDF5, FGF23, FGF19, SEMA6C, IL15** |
| Cluster 3 vs Cluster 1 | **10** | **MSTN, SEMA6B, IFNA10, CCL16, FGF8, TXLNA, PSPN, UCN3, GRN, CKLF** |
| Cluster 3 vs Cluster 2 | **4** | **CMTM5, NTS, BMP8B, INHBC** |
| Cluster 4 vs Cluster 1 | **13** | **BDNF, IL1F10, CMTM8, IL21, SCT, RETNLB, TGFB3, CMTM4, NTF3, SECTM1, IL37, CXCL2, BMP2** |
| Cluster 4 vs Cluster 2 | **8** | **LTBP2, HAMP, CXCL1, CXCL6, SEMA4G, IFNL1, NRG4, ESM1** |
| Cluster 4 vs Cluster 3 | **29** | **CXCL10, CCL8, NMB, IGF1, PLAU, CCL23, AMBN, FGF2, PDGFRL, AREG, GREM1, CXCL9, CCL18, TNFSF14, SEMA6D, AZU1, EPO, CRH, GDF11, BMP7, CXCL8, PYY, CCL25, RABEP2, IL1B, PPBPP2, NRG2, ADIPOQ, SST** |

# Resource Identification Initiative

We identified potential compounds that target AF signature by Connectivity Map (CMap). (CMap, RRID:SCR_016204)

We used WGCNA package to construct coexpression matrix and screen for hub genes. (Weighted Gene Co-expression Network Analysis, RRID:SCR_003302)

We used STRING database to construct protein-protein-interaction network. (STRING, RRID:SCR_005223)

We utilized SHAP method to interpretate the Machine Learning-based diagnostic model. (SHapley Additive ExPlanations, RRID:SCR_021362)

The ssGSEA analysis was performed by using GSVA package. (GSVA, RRID:SCR_021058)

The GSEA analysis was performed by using fgsea package. (fgsea, RRID:SCR_020938)

An XGBoost classification model was used to construct a diagnostic model. (XGBoost, RRID:SCR_021361)

The clusterProfiler package was used for GO/KEGG annotation analysis. (clusterProfiler, RRID:SCR_016884)

The gene sets we used in our study was derived from GEO database. (Gene Expression Omnibus (GEO), RRID:SCR_005012)

The affy package was used for processing expression matrix based on affymatrix array. (affy, RRID:SCR_012835)

Cytoscape software (version 3.9.0) was used for visualization of PPI network. (Cytoscape, RRID:SCR_003032)

MCODE plugin was used for identifying hub gene clusters in PPI network. (MCODE, RRID:SCR_015828)

We used limma package for the analysis of gene expression microarray data. (LIMMA, RRID:SCR_010943)

We used the “CIBERSORT” algorithm to estimate the relative infiltration proportion of 22 immune cell types from AF samples. (CIBERSORT, RRID:SCR_016955)

We used ProtParam and Protscale tool to interpret the physiochemical information of CXCL12. (ProtParam Tool, RRID:SCR_018087); (ExPASy Bioinformatics Resource Portal, RRID:SCR_012880)

TMHMM was used to predict transmembrane helices in CXCL12. (TMHMM Server, RRID:SCR_014935)

We used GeneMANIA to investigate the protein-to-protein interactions among the four hub genes. (GeneMANIA, RRID:SCR_005709)
